# Submuscular Plating, Titanium Elastic Nailing, and External Fixation for Pediatric Femoral Shaft Fractures: A Comparative Systematic Review and Meta-Analysis

**DOI:** 10.7759/cureus.101338

**Published:** 2026-01-12

**Authors:** Ahmed Elnewishy, Sohaib Shah, Pir Zarak Khan, Rami A Abowali, Shajee Ud Din

**Affiliations:** 1 Trauma and Orthopaedics, Royal Berkshire Hospital, Reading, GBR; 2 Trauma and Orthopaedics, Maidstone and Tunbridge Wells NHS Trust, Tunbridge Wells, GBR

**Keywords:** comparative outcomes, external fixation, pediatric femoral shaft fractures, submuscular plating, systematic review and meta-analysis, titanium elastic nailing (tens/esin)

## Abstract

Pediatric femoral shaft fractures are commonly treated with titanium elastic nailing (TENS), submuscular plating (SMP), or external fixation (EF), but the optimal fixation strategy remains controversial. This systematic review and meta-analysis compared perioperative metrics, union, complications, and functional outcomes between SMP, TENS, and EF in skeletally immature patients. A comprehensive search of PubMed, Scopus, Web of Science, Embase, and Google Scholar (up to November 2025) identified comparative studies of diaphyseal pediatric femoral shaft fractures treated with at least two of these modalities. Thirteen studies involving 759 fractures were included: 410 managed with TENS, 246 with submuscular or locking plates, and 89 with EF, plus a small hybrid intramedullary external construct subgroup. Data were pooled using fixed- or random-effects models to calculate standardized mean differences and odds ratios with 95% confidence intervals, and methodological quality was assessed using the Downs and Black checklist. TENS was associated with significantly shorter operative time and lower blood loss than SMP, while hospital stay and time to union were broadly similar. SMP, however, achieved a higher proportion of excellent Flynn functional outcomes and markedly fewer soft-tissue irritation symptoms related to hardware prominence, with no clear differences in infection, unplanned reoperation, implant removal, or radiation exposure. Compared with EF, TENS provided faster or equivalent radiographic union, substantially lower surgical-site and pin-tract infection rates, fewer pin-site problems, and a trend toward reduced refracture after frame or implant removal while maintaining reliable limb alignment and function. Heterogeneity ranged from low to high across outcomes, but no meaningful publication bias was detected. Overall, TENS appears to be an efficient, low-morbidity option for most length-stable pediatric femoral shaft fractures; SMP offers superior stability and functional outcomes in heavier children and length-unstable patterns; and EF should be reserved for severe open injuries, polytrauma, or damage-control scenarios rather than routine femoral shaft fracture management.

## Introduction and background

Pediatric femoral shaft fractures represent one of the most significant long-bone injuries in children, accounting for approximately 1.4-1.7% of all pediatric fractures [1]. Although relatively uncommon, they remain among the most frequent injuries requiring hospitalization because of the associated morbidity, need for surgical intervention, and prolonged rehabilitation [2]. These fractures exhibit a well-described bimodal age distribution. The first peak occurs in children aged two to four years and is most often related to low-energy domestic falls. The second peak occurs in adolescents aged 10-14 years and is typically associated with high-energy mechanisms, such as road-traffic trauma or sports injuries [3,4]. A consistent male predominance of roughly two to one has been reported, likely reflecting greater exposure to high-risk activities [5].

Nearly half of all cases are caused by traffic accidents, followed by falls from height and sports-related injuries [4]. Emerging evidence also suggests that childhood obesity contributes to the risk of femoral shaft fractures because of altered biomechanics and increased ground-reaction forces during impact [4]. Limb overgrowth of 0.8-1.0 cm is common secondary to hyperemia and stimulation of the growth plate during healing, which may influence long-term alignment [3]. Malunion, limb-length discrepancy, and nonunion remain important complications, particularly in unstable fracture patterns or when managed conservatively [5]. Sociodemographic disparities have also been documented, with underrepresented minority children experiencing longer hospital stays and higher complication rates, which suggests inequities in access and quality of care [6].

Titanium elastic nailing (TEN) is one of the most widely used operative modalities for pediatric femoral shaft fractures. Prospective studies consistently demonstrate excellent outcomes, including predictable union. One study reported fracture healing in 21.74% of patients by eight weeks, 56.52% by 10 weeks, and 21.74% by 12 weeks, with more than 90% of children achieving excellent or satisfactory outcomes based on the Flynn criteria [7]. Similar results have been observed in multicenter and long-term cohorts, with union commonly achieved within nine weeks and excellent or good functional outcomes in most patients [8]. TEN has therefore become a reliable and versatile option that performs well even in resource-limited settings [9,10].

For length-unstable or complex fracture patterns, submuscular plating (SMP), also known as submuscular bridge plating, has emerged as an increasingly preferred alternative. Recent systematic reviews support SMP as a safe and effective method, with lower rates of angular deformity, soft-tissue irritation, and radiation exposure, although at the cost of slightly increased intraoperative blood loss [11]. Another meta-analysis of 568 cases reported comparable union and limb-length outcomes between SMP and ESIN, with SMP demonstrating fewer postoperative adverse events [12]. Clinical series reinforce these conclusions. A prospective cohort of 40 children reported 85% excellent outcomes, a mean union time of 7.2 weeks, and no clinically meaningful limb-length discrepancy [13]. Additional studies, including those from South Africa, confirm timely radiographic union, restoration of hip and knee motion, and strong biomechanical stability in length-unstable fractures treated with SMP [14]. High union rates and excellent functional outcomes have also been documented in complex and high-energy fractures, with one study reporting complete union within 11 weeks and 96% excellent or acceptable results [15]. SMP has also been shown to maintain alignment effectively in unstable or comminuted fracture patterns, a finding supported by earlier work [16].

External fixation (EF) continues to play an important role in the treatment of severe open fractures, polytrauma, and highly unstable fracture patterns where rapid stabilization is required. A cohort of 289 pediatric patients demonstrated that EF is effective for complex femoral fractures, achieving reliable union but with notable risks including refracture and minor limb-length discrepancy [17]. Newer approaches, such as ultrasound-guided closed reduction combined with EF, have shown potential for reducing fluoroscopy exposure and early complications. One study involving 19 children aged 5-11 years reported a 100% reduction and healing success rate [18]. Despite these advances, refracture following fixator removal remains a significant limitation. A study of 165 cases identified fracture pattern based on the AO Pediatric Comprehensive Classification of Long Bone Fractures (PCCF) classification and the fixation length ratio L2-L3 as independent predictors of refracture. This highlights the need for cautious removal and optimal frame configuration [19]. Comparative studies indicate that EF and locking plates both provide satisfactory outcomes in length-unstable fractures, although EF is associated with shorter operative times and slightly increased limb-length discrepancy [20]. A recent meta-analysis of 718 patients found that flexible intramedullary nailing results in lower infection, malunion, and deformity rates than EF, supporting the use of EF mainly for open fractures or situations requiring rapid stabilization rather than for routine femoral shaft fractures [21].

Review objective

The objective of this review is to systematically compare the clinical, radiographic, and functional outcomes of SMP, TEN, and EF in the management of pediatric femoral shaft fractures.

Methods

Search Strategy

In December 2025, to identify comparative studies evaluating SMP, TENS/ESIN, and EF for pediatric femoral shaft fractures, a comprehensive literature search was conducted. The search covered PubMed, Scopus, Web of Science, Embase, and Google Scholar, using combinations of Medical Subject Headings (MeSH) and free-text terms, including “pediatric femoral shaft fracture”, “femur fracture”, “titanium elastic nailing”, “elastic stable intramedullary nailing”, “submuscular plating”, “pediatric locking plate”, “external fixation”, and “operative fixation in children”. Boolean operators (“AND,” “OR”) and filters for English-language human studies were applied. To identify additional relevant studies not captured by the electronic search, the reference lists of all eligible articles and related systematic reviews were manually screened to ensure completeness.

Inclusion Criteria

Prospective or retrospective comparative cohorts or randomized controlled trials were eligible for inclusion if they were directly evaluating two or more operative modalities among SMP, TENS, and EF in skeletally immature patients with diaphyseal femoral shaft fractures. Only studies reporting extractable numerical data for at least one postoperative endpoint - such as operative time, blood loss, hospital stay, union time, infection, refracture, soft-tissue irritation, implant removal, unplanned reoperation, or Flynn functional outcomes - were included. For eligibility, full-text studies published in English were required.

Exclusion Criteria

If they did not directly compare the fixation methods of interest, did not report extractable quantitative outcomes, or included mixed fracture types without an isolated pediatric diaphyseal femoral shaft subgroup, studies were excluded. Case reports, single-arm case series, technique notes, cadaveric or biomechanical experiments, narrative reviews, commentaries, and conference abstracts were excluded. To avoid duplication when multiple publications originated from the same patient cohort, the most complete or most recent dataset with the longest follow-up duration was selected.

Outcome Measures

Operative parameters (operative duration, estimated blood loss, fluoroscopy or radiation time, and hospital stay), radiographic measures (time to union), and postoperative complications (infection, refracture, soft-tissue or implant irritation, pin-site problems, implant removal, and unplanned reoperation) were included as the primary outcomes of interest. Functional outcomes, when reported, were extracted using the Flynn criteria and other validated pediatric functional scoring systems. To contextualize treatment effects across studies, demographic and perioperative variables, including patient age, sex, fracture characteristics, surgical technique, and follow-up period, were also collected.

Data Extraction and Quality Assessment

Data extraction was performed independently by two reviewers, using standardized forms to collect information on study design, sample size, patient demographics, fracture characteristics, surgical techniques, follow-up duration, and all reported clinical outcomes. For accuracy, the extracted data were cross-checked, and discrepancies were resolved through consensus. Using the Downs and Black checklist methodological quality was assessed [22], which evaluates reporting quality, internal and external validity, bias, confounding, and statistical power. Each study was assigned a total score from the 28-point scale and categorized as Excellent, Good, Fair, or Poor according to established criteria, and these ratings were incorporated into the interpretation of pooled findings.

Statistical Analysis

Using Review Manager (RevMan v5.4, The Cochrane Collaboration, London, UK), all statistical analyses were conducted [23]. For continuous outcomes, results were pooled as standardized mean differences (SMD) with 95% confidence intervals (CI). For dichotomous outcomes, odds ratios (OR) with 95% CI were calculated. Fixed-effect and random effect models were selected for analyses based on methodological consistency across studies. Using the chi-square (chi²) test and the I² statistic, statistical heterogeneity was evaluated. By visual inspection of funnel plots, potential publication bias was assessed, and when enough studies were available, by performing Egger’s regression test, with a p-value < 0.05 considered indicative of small-study effects.

Results

Search Results and Study Selection

Across PubMed, Scopus, Web of Science, Embase, and Google Scholar, a comprehensive systematic search was performed to identify comparative studies evaluating SMP, TEN/ESIN, and EF for the management of pediatric femoral shaft fractures. The search encompassed publications up to November 2025. Combinations of keywords and MeSH terms included in search terms such as “pediatric femoral shaft fracture”, “titanium elastic nailing”, “submuscular plating”, “external fixation”, “elastic stable intramedullary nailing”, “diaphyseal femur fracture”, “operative management”, and “clinical outcomes”. Boolean operators (“AND,” “OR”) and filters for English-language human studies were applied. To ensure complete coverage, reference lists of included articles and relevant systematic reviews were manually screened.

There are 612 records, of which 148 duplicates were removed through the initial search. Title and abstract screening excluded 397 studies due to irrelevance, non-comparative design, absence of operative cohorts, or evaluation of non-shaft fracture patterns. Full-text assessment was conducted on 67 articles, with exclusion of studies that did not directly compare at least two of the three operative modalities (TEN/ESIN, SMP, EF), lacked extractable numerical outcomes, included pathological or metabolic conditions, or were biomechanical/cadaveric experiments. Thirteen studies were included in the final review and quantitative synthesis.

With disagreements resolved through consensus, two reviewers independently screened all titles, abstracts, and full-text manuscripts. Using a standardized form, data extraction was performed, capturing study design, demographic characteristics, fracture morphology, surgical technique, perioperative variables, union metrics, functional scores, and complication profiles.

**Figure 1:**
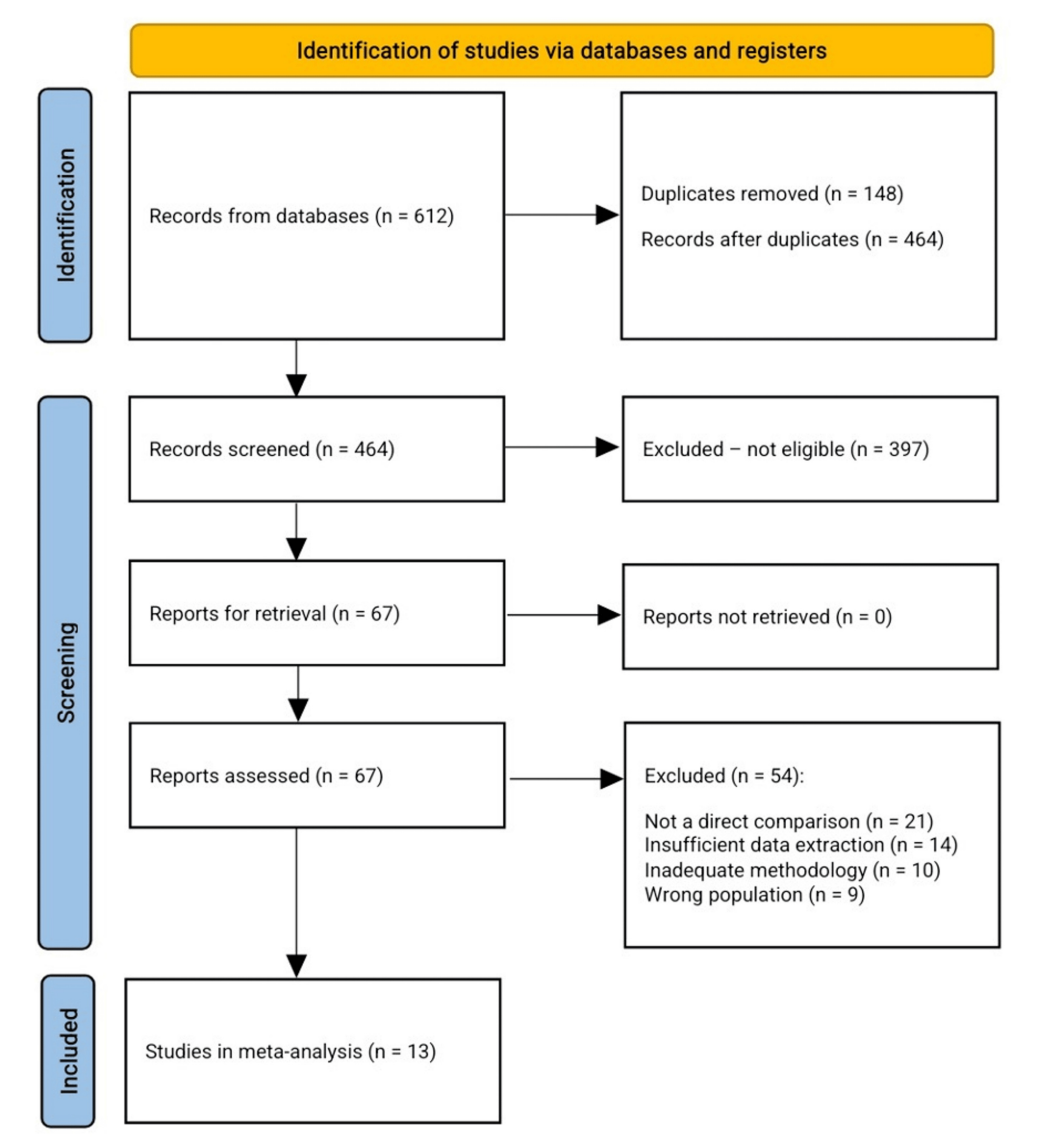
PRISMA flowchart for the included studies. PRISMA: Preferred Reporting Items for Systematic Reviews and Meta-Analyses

The study selection process is presented according to the Preferred Reporting Items for Systematic Reviews and Meta-Analyses (PRISMA) guidelines in Figure 1.

Study Characteristics

A total of 759 pediatric femoral shaft fractures were analyzed: 410 treated with TEN/ESIN, 246 with submuscular or pediatric locking plates, 89 with EF, and 14 with a hybrid IOLE construct across 13 comparative cohorts. Most cohorts were single-center level III studies, with three level II randomized trials. Patients were predominantly 5-15 years old, with mean ages of 7-10 years and body weights of 25-40 kg. Displaced diaphyseal fractures, subsets examined length-unstable or distal third patterns, high-grade open injuries, or complex morphologies were involved. One multicenter series incorporated the hybrid IOLE device.

Sized to ~40% canal diameter, dual retrograde TEN/ESIN nails were used, sometimes followed by four to six weeks of spica or bracing in unstable fractures. Through two small lateral incisions, SMP applied low-profile or locking plates, with earlier series grouping open and submuscular approaches. For open fractures, polytrauma, or very young patients, with fixator removal at 6-12 weeks, monolateral or hybrid frames EF was favored. To improve control of length, rotation, and alignment, IOLE combined dual intramedullary nails with an external fixator, and alignment. Follow-up was generally ≥12 months, commonly two to five years; some series focused on short-term (12-week) radiographic and functional endpoints.

Across studies, outcomes were consistent. Surgical time, estimated blood loss, fluoroscopy exposure, and hospital stay (with or without implant removal) are included as operative metrics. Time to union is typically within 7-13 weeks, extending to four to six months in cohorts using stricter criteria. Alignment parameters and limb-length discrepancy were routinely assessed. Functional evaluation relied primarily on Flynn scores, with some reporting Harris Hip Score (HHS), Knee Society Score (KSS), Pediatric Quality of Life Inventory (PedsQL), or Pediatric Outcomes Data Collection Instrument (PODCI) at up to two years. Additionally, >90% excellent/satisfactory Flynn ratings and reliable union, with rare nonunion or limb-length discrepancy >2 cm, were achieved by TEN and SMP. Though EF demonstrated more variability in early weight bearing and soft-tissue tolerance, EF and IOLE also showed high union rates.

Complication profiles were well documented. Loss of reduction, refracture, deep infection, or unplanned reoperation, representing major complications, were low (0-5% across groups) but slightly higher with EF, especially refracture after frame removal. TEN/ESIN had the highest frequency of minor hardware irritation, with nail prominence or soft-tissue symptoms reported in up to ~40% of cases. Operative times and greater blood loss but fewer hardware complaints were involved in SMP longer, and in several studies produced higher proportions of “excellent” Flynn outcomes and earlier restoration of motion and weight bearing than TEN. The shortest operative times and lowest blood loss offered by EF, with immediate or early weight bearing, but also the highest pin-tract infection rates, poorer cosmesis, and increased refracture. With lower malalignment and complication rates, IOLE demonstrated similar or slightly faster union and earlier weight bearing than TEN or EF. Table 1 summarizes the study designs, demographics, interventions, follow-up, outcomes, and principal findings.

**Table 1:** Summary of the study characteristics, including sample size, patient demographics, interventions, follow-up duration, complications, and primary outcomes for studies comparing submuscular plating (SMP), titanium elastic nailing (TEN), and external fixation (EF) in pediatric femoral shaft fractures. TEN: Titanium Elastic Nailing; ESIN/FN: Elastic Stable Intramedullary Nailing/Flexible Nailing; SMP: Submuscular Plating; SBP: Submuscular Bridge Plating; EF: External Fixation; IOLE: Intramedullary Osteosynthesis Linked External-Fixator; LOS: Length of Stay; ROM: Range of Motion; WB: Weight-Bearing; LLD: Limb-Length Discrepancy; VAS: Visual Analog Scale; PODCI: Pediatric Outcomes Data Collection Instrument; HHS: Harris Hip Score; KSS: Knee Society Score; PedsQL: Pediatric Quality of Life Inventory; RUSH: Radiographic Union Score for Hip (adapted); NUSS: Non-Union Scoring System

Category	Study Design	Sample Size (TEN / SMP / EF)	Level of Evidence	Patient Demographics	Intervention Details (Groups)	Follow-up Duration	Outcome Measures	Results	Complications	Conclusion
Aditya et al. [24]	Prospective comparative, quasi-randomized (single center)	22 TEN / 18 SMP / 0 EF (40 analyzed; 44 initially)	II	Age 5–13 yrs; mean 8.95±2.49 (TEN) vs 11.22±1.95 (SMP); ≈80% male; mix of transverse/spiral/oblique/comminuted; length-stable: 12/22 TEN, 10/18 SMP; length-unstable: 10/22 TEN, 8/18 SMP	TEN (ESIN): dual elastic nails; SMP: minimally invasive submuscular bridge plating	Clinical FU ≈12.7±1.2 months; minimum 12 months; radiologic until union	Operative time, incision length, EBL, fluoroscopy, LOS, union time (weeks), VAS pain, analgesic days, Flynn score	Union: 11.18±4.35 wks (TEN) vs 9.67±5.32 wks (SMP). Flynn: TEN ≈72.7% excellent, 18.2% satisfactory, 9.1% poor; SMP 94.4% excellent, 0% satisfactory, 5.6% poor (p≈0.036). TEN had shorter operative time, smaller incision, less blood loss (exact mins/mL not reported in my extraction).	TEN: 1 non-union with implant failure + refracture; 2 malunion; 3 (~13.6%) nail irritation; 1 deep infection; 1 shortening <2 cm. SMP: 1 deep infection; 1 malunion; 1 implant failure; 1 shortening <2 cm.	Both methods effective. TEN = less invasive, shorter surgery, more minor hardware problems; SMP = slightly earlier union & better Flynn distribution.
Allen et al. [25]	Retrospective cohort (single level-1 trauma center, 2004–2014)	50 TEN / 15 Plate (open+SMP) / 0 EF	III	63 pts, 65 fractures; mean age 8.7±2.0 yrs; mean weight 33.9±10.5 kg; ~66% male; high-energy trauma common; 21 length-unstable fractures (14 TEN, 7 plate); 4 open fractures (all TEN)	TEN: titanium elastic nails. Plate: open plating + submuscular plating analyzed together	FU to union and through elective hardware removal (time not fixed, but typically >6–12 months)	Op time, EBL, fluoroscopy, LOS, VAS change, opioid use, Flynn score, cost (implant + anesthesia), same variables at removal	Index surgery: Op time 1.6±1.1 h (TEN) vs 2.5±0.9 h (plate) (*p*=0.007). EBL 40.1±56.6 mL vs 79.0±67.9 mL (*p*≈0.06). LOS 3.7 vs 3.5 days (NS). Flynn: TEN 44/50 (88%) excellent, 4 (8%) satisfactory, 2 (4%) poor; Plate 13/15 (87%) excellent, 0 satisfactory, 2 (13%) poor (similar). Removal: Op time 0.9 h (TEN) vs 1.33 h (plate) (*p*=0.02); EBL 13.3 vs 50.4 mL (*p*=0.01); fluoro 0.14 vs 0.56 min (*p*=0.04).	No deep infections or hardware failures reported. TEN: poor outcomes due to malunion or LLD <2 cm; Plate: 2 poor outcomes (LLD >2 cm and procurvatum >10°). No reoperations planned for poor results (one lost to FU).	TEN and plates both good; TEN gives similar outcomes with shorter surgery, less blood loss and anesthesia time, lower overall cost.
Andreacchio et al. [26]	Retrospective two-center comparison (Italy + France)	23 ESIN / 0 SMP / 15 EF	III	Children ≤8 yrs; ESIN: 5.6 yrs, 17 boys/6 girls, wt 20.2 kg; EF: 5.9 yrs, 8 boys/7 girls, wt 18.7 kg; all closed fractures; 1 polytrauma in EF vs 8 in ESIN	ESIN: dual retrograde nails; EF: unilateral external fixator. All fractures AO 32D4.1/4.2/5.1/5.2	Mean FU 60.4 months (ESIN) vs 67 months (EF); at least 1 year after full healing; hardware removed at 2–4 mo (EF) and 7 mo (ESIN)	Time to partial/full WB, time to healing (months), ROM, LLD, alignment, Flynn score (ESIN)	Partial WB: 2.9 days (EF) vs 30.9 days (ESIN) (*p*<0.001). Full WB: 34.5 days (EF) vs 58.8 days (ESIN) (*p*<0.001). Healing: 5.1 mo (EF) vs 4.2 mo (ESIN) (*p*=0.002). At final FU all pain-free, full ROM, no LLD >1 cm. Flynn ESIN: 22/23 (95.5%) excellent, 1 satisfactory.	EF: 2/15 pin-site infections (13.3%), 1/15 refracture (6.7%) after frame removal. ESIN: 1/23 (4.3%) nail migration needing 2 further ops. Overall complications 26.7% EF vs 4.3% ESIN (*p* ns).	Both suitable <8 yrs. EF allows very early WB but more pin-site problems and refracture; ESIN heals quicker and fewer complications.
Aslani et al. [27]	Retrospective comparative (single trauma center)	13 TEN / 0 SMP / 14 EF (open Gustilo IIIA/B only)	III	Children <12 yrs with high-grade open femoral shaft fractures; group age/sex similar; most due to high-energy MVAs	TEN (±extra pin): flexible nails; EF: unilateral external fixator; all debrided within 6 h; IV cefazolin+gentamicin±penicillin	FU ≥1 year, some up to 2 yrs; EF removed at ≈2.5 months	Union time (months), infection, malunion, refracture, LLD (>1 cm), knee ROM, time to walking & return to school	Union: 3.89 mo (EF) vs 3.61 mo (TEN) (NS). Walking allowed 4–10 days in both; EF often PWB/FWB, TEN often NWB/PWB initially. Normal knee ROM: 20 wks (EF) vs 10 wks (TEN). Return to school: 12 wks (EF) vs 4 wks (TEN) (TEN significantly faster). LOS ~8 days EF vs 6.5 days TEN (approx).	EF: 4/14 (28.5%) pin-tract infections (needed pin change), 2/14 (14.2%) refractures, 1 LLD >1 cm. TEN: 1/13 (7.7%) bursitis at entry site; 0 refracture, 0 LLD >1 cm. Combined TEN+pin subgroup (5 pts) had no complications.	Both methods achieve union; TEN (esp. TEN+pin) gives faster ROM and return to school and fewer complications compared with EF.
Bisaccia et al. [28]	Retrospective multicenter (Italy, Spain, Russia)	22 TEN / 0 SMP / 22 EF + 14 IOLE	III	Age range 6–14 yrs; mean age ≈7.2 (TEN), 9.4 (EF), 11.5 (IOLE); similar sex ratios; AO 32 A/B/C patterns; NUSS scores highest in IOLE	TEN: standard elastic nails; EF: external fixator; IOLE: two TENs linked to Hoffman II external frame (hybrid)	Mean FU ≈14.2 months in all groups; implants removed ≈148–160 days post-op	VAS, HHS, KSS, PedsQL, time to union (days), RUSH score, WB progression	Healing time: ≈139 days (TEN), 140 (EF), 128 (IOLE) (NS overall). IOLE had higher RUSH scores, earlier WB (significantly shorter non-WB time). Early (1–3 mo) HHS/KSS/PedsQL worse in TEN than EF/IOLE; by 12 mo all groups similar.	Complications: TEN 10/22 (45.5%), EF 9/22 (40.9%), IOLE 4/14 (28.6%). Malalignment (>15°) and rotational deformity seen in TEN/EF, none in IOLE. Superficial infections and blood loss issues scattered among groups; pain at nail entry more common in TEN.	IOLE hybrid has lowest malalignment and lowest overall complication rate, with union and function comparable or superior to TEN/EF and earlier WB.
Chen et al. [29]	Retrospective single-surgeon cohort	28 TEN (FNs) / 30 SMP / 0 EF	III	Mean age 7.7±2.0 yrs (SMP 8.1±2.4; FN 7.3±2.0); 26% female; mixed fracture patterns: 21 comminuted, 16 transverse, 13 oblique, 8 spiral; mechanism: ~47% recreational, 43% MVA	TEN (FN): flexible nails; SMP: submuscular bridge plating; surgeon tended to use SMP for comminuted, FN for transverse	Mean clinical FU 22±28 mo; radiologic FU 5.3±5.4 mo; 47/58 had implant removal (24 SMP, 23 FN) at ~197 days	Index and removal: EBL, op time, LOS, infection, implant irritation, malunion, non-union, LLD >2 cm	Adjusted analysis: Op time insertion: FN shorter by 24 min (*p*<0.001). EBL insertion: FN lower by 38 mL (*p*<0.001). Op time removal: FN shorter by 15 min (*p*<0.001). LOS after insertion 0.2 days shorter with FN (*p*≈0.03). EBL at removal lower (median 0 vs 75 mL; *p*=0.08). Alignment and union radiographically normal in all cases at 5.3 mo.	Insertion/removal: Infection: 4/30 SMP vs 3/28 FN (all resolved). Implant irritation: 2 SMP vs 7 FN. No AVN, HO, LLD >2 cm, malunion, non-union in either group.	Both effective; TEN/FN gives shorter ops, less blood loss, shorter LOS with similar union and alignment; SMP has fewer nail-prominence symptoms but more infections.
El-Adly et al. [30]	Prospective randomized controlled trial (single center)	25 TEN / 25 SMP / 0 EF	II	Age 6–12 yrs; mean 7.96±1.6 (TEN) vs 8.28±1.6 (SMP); TEN 88% male vs SMP 72% male; mechanisms: fall from height ~36–44%, MVAs, falls on ground, motorcycles, stairs; mostly closed fractures	Group 1 – TEN: flexible IMN (nail size ≈40% canal). Group 2 – SMP: submuscular locked plate using cluster technique	FU minimum 12 months; visits at 2 wks, 6 wks, 3 mo, then every 3 mo	Trauma-to-surgery time, op time, EBL, radiation time, LOS, union (months), WB at 1 mo, knee ROM at 6 & 12 mo, Flynn score	Trauma→surgery: 0.8±0.9 days (TEN) vs 1.2±1.1 (SMP). Op time: 45.4±10.5 vs 60±11.5 min (NS but longer for SMP). EBL: 12.44±4.7 mL (TEN) vs 67.56±16.7 mL (SMP) (*p*=0.05). Union: 6.2±0.4 mo (TEN) vs 6.3±0.2 mo (SMP). WB at 1 mo: 17/25 (68%) TEN vs 23/25 (92%) SMP (*p*=0.03). Limited knee ROM at 6 mo: 4/25 (16%) TEN vs 0/25 SMP (*p*=0.02); persisted at 12 mo. Flynn: TEN 76% excellent, 24% satisfactory; SMP 92% excellent, 8% satisfactory.	TEN: 4/25 (16%) nail-entry irritation with painful knee and limited ROM; no infections, no failures. SMP: fewer malalignments and smaller mean LLD (0.75 vs 1.25 cm); no infections or major hardware problems recorded.	TEN: much less blood loss, slightly shorter op time; SMP: earlier WB, better ROM, more excellent Flynn outcomes, fewer residual deformities.
Hayat et al. [31]	Prospective randomized clinical trial (single center)	51 TEN / 51 SMP / 0 EF	II	Age 6–12 yrs, mean 9.05±1.69; TEN 8.82±1.62, SMP 9.27±1.74 (*p*=0.179); 76.5% male overall	Group A – TEN: titanium elastic nails. Group B – SMP: submuscular locking plates	FU at 1, 3, 6, 12 weeks only (short-term)	Infection, implant failure, radiological union (Hammer), hip function by Flynn	Union at 6 wks: 47/51 (92.2%) TEN vs 48/51 (94.1%) SMP (NS). Union at 12 wks: 49/51 (96.1%) TEN vs 51/51 (100%) SMP (NS). Flynn at 12 wks: TEN – 37/51 (72.5%) excellent, 13 (25.5%) good, 1 (2%) fair; SMP – 47/51 (92.2%) excellent, 4 (7.8%) good, 0 fair (*p*=0.031). Earlier weeks also favored SMP for better function.	TEN: 3/51 (5.9%) superficial infection; 2/51 (3.9%) delayed union. SMP: 1/51 (1.9%) deep infection; no delayed union; no implant failure in either group.	Both give similar union; SMP shows better early functional outcomes, fewer minor infections and delayed unions within 12 weeks.
James et al. [32]	Open-label RCT with concealed allocation (multi-center in South India)	20 ESIN / 20 SMP / 0 EF	II	Age 6–15 yrs; mean 9.45±3.1 (ESIN) vs 10.2±2.7 (SMP); almost all <50 kg; mechanisms mainly road traffic accidents (20/20 ESIN, 17/20 SMP)	ESIN: TENS sized 0.4× canal, retrograde. SMP: titanium LCP submuscular bridge plating	FU minimum 24 months; assessments at 2, 6, 24 months	Primary: adverse surgical events. Secondary: union time (wks), malunion, LLD, PODCI at 24 mo, op time, EBL, radiation, LOS, cost, rate of implant removal	Adverse events: 5 ESIN vs 3 SMP. Union: 13.05 (8–36) wks ESIN vs 11.95 (8–15) wks SMP (NS). PODCI (sports/physical) at 24 mo: 47.3 (26–56) ESIN vs 51.2 (41–56) SMP (difference < MCID). Anesthesia time: ≈100 vs 105 min; EBL: 60 vs 75 mL (NS). LOS: 4.4 vs 5.4 days (*p*=0.057). Cost: slightly higher ESIN initial bill, but far fewer removals.	ESIN: 2 implant failures (undersized nails) needing revision/cast; 2 painful prominences; 1 genu valgum; 2 LLD 1–1.5 cm. SMP: 1 deep infection needing debridement/re-plating; 1 painful screw; 1 genu valgum; 1 LLD 1.5 cm. Implant exit: 3/20 ESIN vs 15/20 SMP (*p*<0.001).	ESIN and SMP have comparable union, alignment, and 2-yr function; SMP slightly better radiographically but requires many more reoperations, so ESIN preferred in low-resource settings.
Li et al. [33]	Retrospective single center (length-unstable fractures only)	77 ESIN / 45 SMP / 0 EF	III	Age 5–11 yrs, mean 8.1 (ESIN) vs 8.0 (SMP); weight 27.0±5.4 vs 27.0±6.1 kg; similar sex distribution	ESIN: flexible nails + post-op spica/brace 4–6 wks. SMP: submuscular plating + immobilization 2–3 wks	FU >24 months; implant removal 6–14 mo for both groups	Op time, EBL, fluoro shots, LOS, union, angulation, LLD, Flynn, major vs minor complications; same at removal	Index surgery: Op time: 54.5±8.1 (ESIN) vs 73.7±9.7 min (SMP). EBL: 51.7±18.9 vs 106.4±26.6 mL. LOS: 4.0±0.9 vs 5.9±0.8 days (all *p*<0.001). Fluoroscopy ~21 shots both. Flynn: ESIN excellent 54.5% (42/77), excellent+satisfactory 98.7%; SMP excellent 88% (40/45), excellent+satisfactory 97.8%. Removal: ESIN op time 21.1±5.6 vs 49.3±6.3 min; EBL 19.6±6.6 vs 50.9±6.9 mL; LOS 2.9±0.9 vs 3.8±0.8 days (all *p*<0.001).	Major: ESIN 1/77 (1.3%) loss of reduction; SMP 1/45 (2.2%) refracture (conservative). Minor: ESIN 31/77 (40.3%) implant prominence + 4/77 (5.2%) mild angulation; SMP 4/45 (8.9%) implant prominence. No non-union in either group.	ESIN much faster, less blood loss, shorter stay (both index and removal) but more minor hardware complaints; SMP more “excellent” Flynn and fewer minor issues. Both safe for length-unstable fractures.
Li et al. [34]	Retrospective (distal third femoral shaft fractures)	33 ESIN / 0 SMP / 38 EF	III	Age 5–11 yrs, mean 8.0 (ESIN) vs 8.3 (EF); weight 29.0±5.8 vs 29.9±6.6 kg; similar sex and side distribution	ESIN: retrograde nails + spica or cast 4–6 wks. EF: hybrid external fixator + slab 3–4 wks; ExFix removed 6–12 wks, then brace	FU >24 months; ESIN removed in theatre at 4–7 mo; ExFix removed as outpatient at 6–12 wks	Op time, EBL, fluoro, LOS, VAS pain, union, LLD, angulation, Flynn, major/minor complications	Op time: 57.8±11.3 min (ESIN) vs 45.4±7.8 (EF) (*p*<0.01). EBL: 16.4±6.5 vs 9.9±3.5 mL (*p*<0.01). Fluoroscopy 15.5±3.2 vs 13.9±2.4 (*p*=0.02). LOS 4.0±0.8–0.9 (NS). Flynn: ESIN 30/33 (90.9%) excellent, 3 satisfactory; EF 34/38 (89.5%) excellent, 4 satisfactory; no poor in either group. Mean LLD 2.5±1.6 mm (ESIN) vs 4.2±2.8 mm (EF) (*p*<0.01).	Major: ESIN 0; EF 2/38 (5.3%) refractures after frame removal. Minor: ESIN 12/33 (36.4%) implant irritation; 1/33 (3%) infection; 2/33 (6.1%) scar concern. EF 28/38 (73.7%) implant irritation; 18/38 (47.4%) pin-site infection; 9/38 (23.7%) scar concern.	Both achieve good outcomes; EF shorter, less blood loss, no second op for removal but far more pin-site problems, scar issues, and larger LLD; ESIN preferred where resources allow.
Milligan et al. [35]	Retrospective single-center cohort	14 TEN / 14 Plate / 0 EF	III	28 pts; 75% male; mean age 9.7±1.9 yrs (TEN) vs 7.7±1.8 yrs (plate) (*p*=0.008); isolated diaphyseal fractures	TEN (TENS): elastic nails. Plate: submuscular bridge plating. Only fractures deemed suitable for either method included	Minimum 2 yrs, mean 5.3±2.5 yrs (TEN) and 5.3±2.4 yrs (plate); union assessed at 12 wks	LOS, analgesia (24–48 h, mg/kg), time to full WB, union at 12 wks, Flynn	LOS: 7.8±3.0 days (TEN) vs 6.3±2.1 (plate) (*p*=0.134). Analgesia 48 h: 1.12±0.97 mg/kg (TEN) vs 0.82±0.45 (plate) (*p*=0.316). Union at 12 wks: 10/14 TEN vs 14/14 plate (*p*=0.098). Flynn: TEN 6 excellent, 7 satisfactory, 1 poor; plate 10 excellent, 3 satisfactory, 1 poor (*p*=0.32).	TEN: 4 unplanned reoperations before union (2 nail migrations, 1 irritation, 1 end-cap issue); 11 later routine removals. Plate: 0 unscheduled reoperations; 12 elective removals; 1 overgrowth/LLD needing contralateral epiphysiodesis at 12 mo.	Both effective with >90% good/excellent. Trend in favor of plates for LOS, analgesia, union at 12 wks and fewer unplanned returns.
Yigit et al. [36]	Retrospective comparative (single center; midterm + radiation)	32 TEN / 28 SMP / 0 EF	III	Age 6–16 yrs, mean 10.3 yrs; mean weight 39.4 kg (28–55); SBP group: 28 pts (18M/10F); TEN group: 32 pts (18M/14F); 63.3% midshaft, 26.6% proximal, 20.3% distal; AO types A (63.3%), B (33.3%), C (13.3%)	TEN: elastic nails (group 2). SBP: submuscular bridge plating (group 1). All under GA with fluoroscopy	Mean FU 29.8 months (range 12–55); visits at 2, 4, 6, 8, 12 wks then periodically	Op time, EBL, LOS, union (wks), time to full WB, knee ROM, Flynn, fluoroscopy time (sec), DAP (cGy·cm²), estimated lifetime cancer & hereditary risk	Overall union time 7.4 wks (6–10); WB from that point; full ROM ~12 wks. By Flynn: 50/60 (83.3%) excellent, 4 (6.6%) good, 6 (10%) poor. Subgroups: SBP 24 excellent, 2 good, 2 poor; TEN 16 excellent, 2 good, 4 poor. SBP vs TEN: LOS 2.9±0.2 vs 3.1±0.2 days; op time 65.5±2.6 vs 59.0±3.3 min; union 7.0±0.3 vs 7.8±0.2 wks; EBL 112.1±13.7 vs 93.7±13.7 mL (no statistically emphasized differences). Radiation: fluoro time ≈34.7±2.6 s (SBP) vs 40.8±3.0 s (TEN); DAP ≈60.4 vs 70.9 cGy·cm².	No major complications: no non-union, delayed union, deep infection, refracture, compartment syndrome, knee ankylosis in either group. Flynn “poor” outcomes (6 total) related to alignment/LLD thresholds but not requiring major surgery in the reported series.	Both TEN and SBP safe/effective with high excellent rates and similar union; emphasis on minimizing radiation exposure for both patients and staff when using fluoroscopy-dependent techniques.

Quality Assessment of the Included Studies

The Downs and Black ratings were explicitly incorporated into both the quantitative and qualitative synthesis of this review. Studies rated Excellent or Good were prioritized for inclusion in the primary meta-analysis, whereas studies rated Fair or Poor were retained for descriptive comparison and sensitivity analyses. Pooled analyses were repeated after exclusion of lower-quality studies when statistical heterogeneity was substantial or when outcome estimates appeared inconsistent, to verify the stability and robustness of effect estimates. Ensuring that risk-of-bias assessments directly informed data synthesis, weighting, and the overall interpretation of findings, the narrative interpretation also emphasized results supported predominantly by higher-quality evidence.

Moderate methodological quality demonstrated by the included studies, reflecting the mixture of randomized controlled trials and retrospective comparative cohorts typical of pediatric orthopedic trauma research. Most studies performed well in the reporting and internal validity-bias domains; however, notable variability was observed in external validity and especially in the control of confounding, consistent with the predominance of retrospective non-randomized designs. One study was classified as Excellent, four were Good, seven were Fair, and one was rated Poor based on total Downs and Black scores. A full breakdown of scoring across all methodological domains is provided in Table 2.

**Table 2:** Quality assessment of the included studies using the Downs and Black checklist. Scores reflect methodological performance across five key domains with ratings categorized as Excellent (≥25), Good (21–24), Fair (17–20), and Poor (<17).

Study (Citation)	Study Design	Reporting (0–11)	External Validity (0–3)	Internal Validity – Bias (0–7)	Internal Validity – Confounding (0–6)	Power (0–1)	Total Score (max 28)	Quality Rating
Aditya et al. [24]	Prospective quasi-randomized trial	9	2	5	3	0	19	Fair
Allen et al. [25]	Retrospective comparative cohort	9	2	5	4	0	20	Fair
Andreacchio et al. [26]	Retrospective comparative (ESIN vs EF)	8	2	5	3	0	18	Fair
Aslani et al. [27]	Retrospective comparative (open fractures)	7	1	4	3	0	15	Poor
Bisaccia et al. [28]	Retrospective multicenter cohort	9	2	5	4	0	20	Fair
Chen et al. [29]	Retrospective single-surgeon cohort	10	2	6	4	0	22	Good
El-Adly et al. [30]	Prospective randomized controlled trial	10	2	6	5	0	23	Good
Hayat et al. [31]	Randomized clinical trial	9	2	5	5	0	21	Good
James et al. [32]	Randomized controlled trial with allocation concealment	11	3	6	5	0	25	Excellent
Li et al. [33]	Retrospective cohort (length-unstable)	10	2	6	5	0	23	Good
Li et al. [34]	Retrospective cohort (distal third; ESIN vs EF)	9	2	5	4	0	20	Fair
Milligan et al. [35]	Retrospective comparative cohort	8	2	5	3	0	18	Fair
Yigit et al. [36]	Retrospective comparative cohort	9	2	5	4	0	20	Fair

## Review

Results of the meta-analysis

Comparison of Operative Time Between SMP and TENS

**Figure 2:**
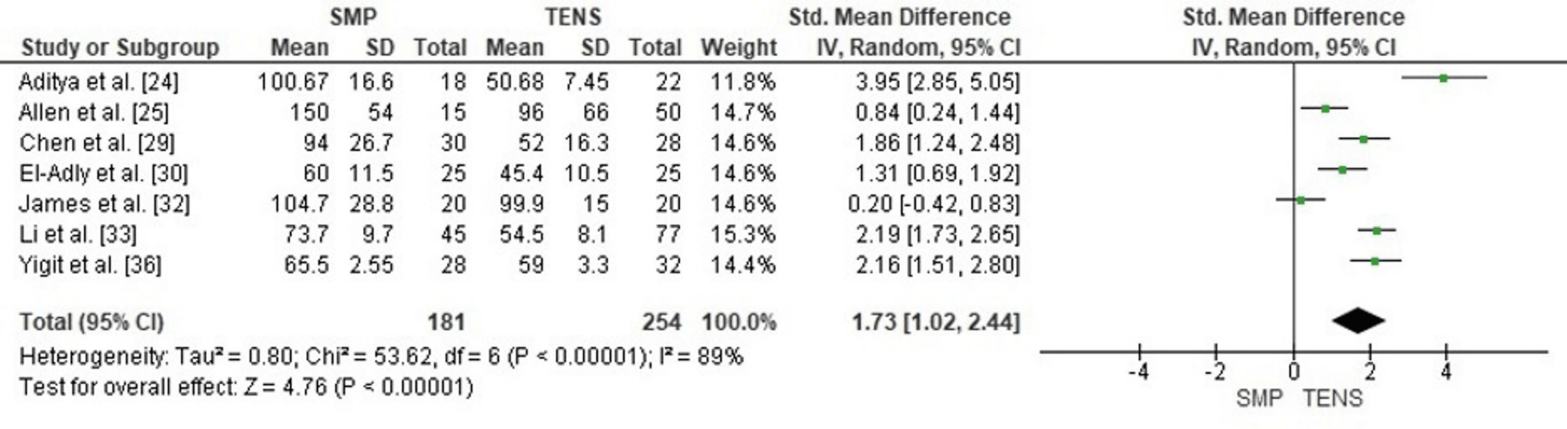
Forest plot comparing operative time between submuscular plating and titanium elastic nailing. SMD: Standardized Mean Difference; CI: Confidence Interval Source: Refs [24,25,29,30,32,33,36]

A pooled comparison of operative duration demonstrated that SMP was associated with significantly longer operative time compared with TENS (SMD = 1.73; 95% CI: 1.02-2.44; p < 0.00001). Heterogeneity was substantial (chi² = 53.62, df = 6, p < 0.00001; I² = 89%), reflecting variability in fracture patterns, surgical approaches, and surgeon experience across the included studies (Figure 2). Despite this variability, all individual studies favored shorter operative times with TENS.

Publication Bias Assessment

**Figure 3:**
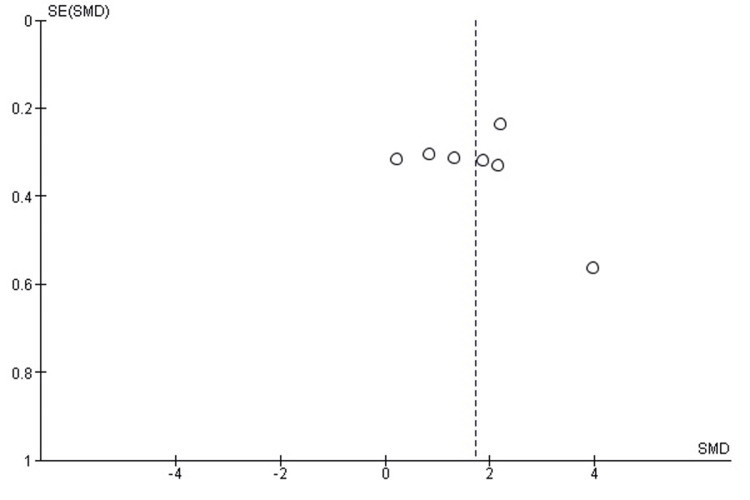
Funnel plot assessing publication bias for operative time comparison. SE: Standard Error; SMD: Standardized Mean Difference

The funnel plot (Figure 3) demonstrated mild asymmetry, likely related to variation in sample sizes and operative-technique reporting. However, the distribution did not suggest systematic publication bias, and Egger’s regression test was not statistically significant (p > 0.05), indicating that small-study effects are unlikely to have influenced the pooled results.

Comparison of the Estimated Blood Loss Between SMP and TENS

**Figure 4:**
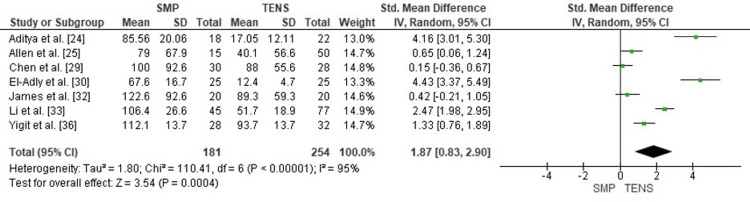
Forest plot comparing estimated blood loss between submuscular plating and titanium elastic nailing. SMD: Standardized Mean Difference; CI: Confidence Interval Source: Refs [24,25,29,30,32,33,36]

Estimated intraoperative blood loss was significantly higher with SMP than with TENS (SMD = 1.87; 95% CI: 0.83-2.90; p = 0.0004). Heterogeneity was considerable (chi² = 110.41, df = 6, p < 0.00001; I² = 95%), indicating marked variability in surgical exposure, use of tourniquets, transfusion thresholds, and documentation of blood loss (Figure 4). Nevertheless, all individual studies favored reduced blood loss with TENS, supporting a consistent advantage of the less invasive technique.

Publication Bias Assessment

**Figure 5:**
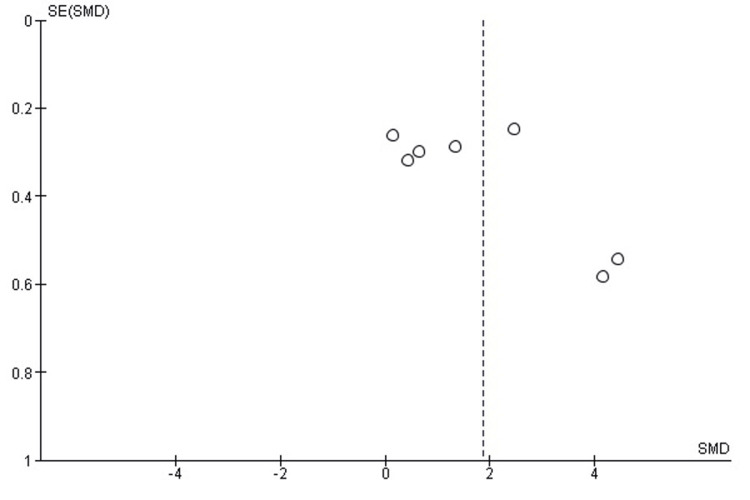
Funnel plot assessing publication bias for estimated blood loss comparison. SE: Standard Error; SMD: Standardized Mean Difference.

The funnel plot (Figure 5) displayed asymmetry, which may be related to small-sample effects or varying reporting quality across studies. However, no strong directional bias was evident, and Egger’s regression test was not statistically significant (p > 0.05), suggesting that substantial publication bias is unlikely for this outcome.

Comparison of Time to Union Between SMP and TENS

**Figure 6:**
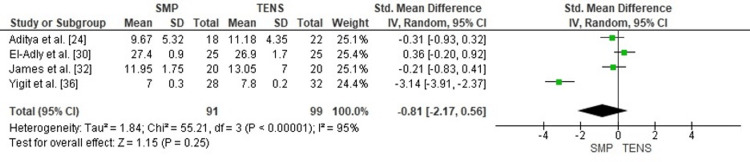
Forest plot comparing time to union between submuscular plating and titanium elastic nailing. SMD: Standardized Mean Difference; CI: Confidence Interval Source: Refs [24,30,32,36]

A pooled comparison of time to radiographic union demonstrated no significant difference between SMP and TENS (SMD = -0.81; 95% CI: -2.17 to 0.56; p = 0.25). Heterogeneity was marked (chi² = 55.21, df = 3, p < 0.00001; I² = 95%), likely reflecting differences in fracture configuration, definition of union, and follow-up intervals (Figure 6). These findings indicate that both fixation methods achieve broadly similar union times across the available studies.

Publication Bias Assessment

**Figure 7:**
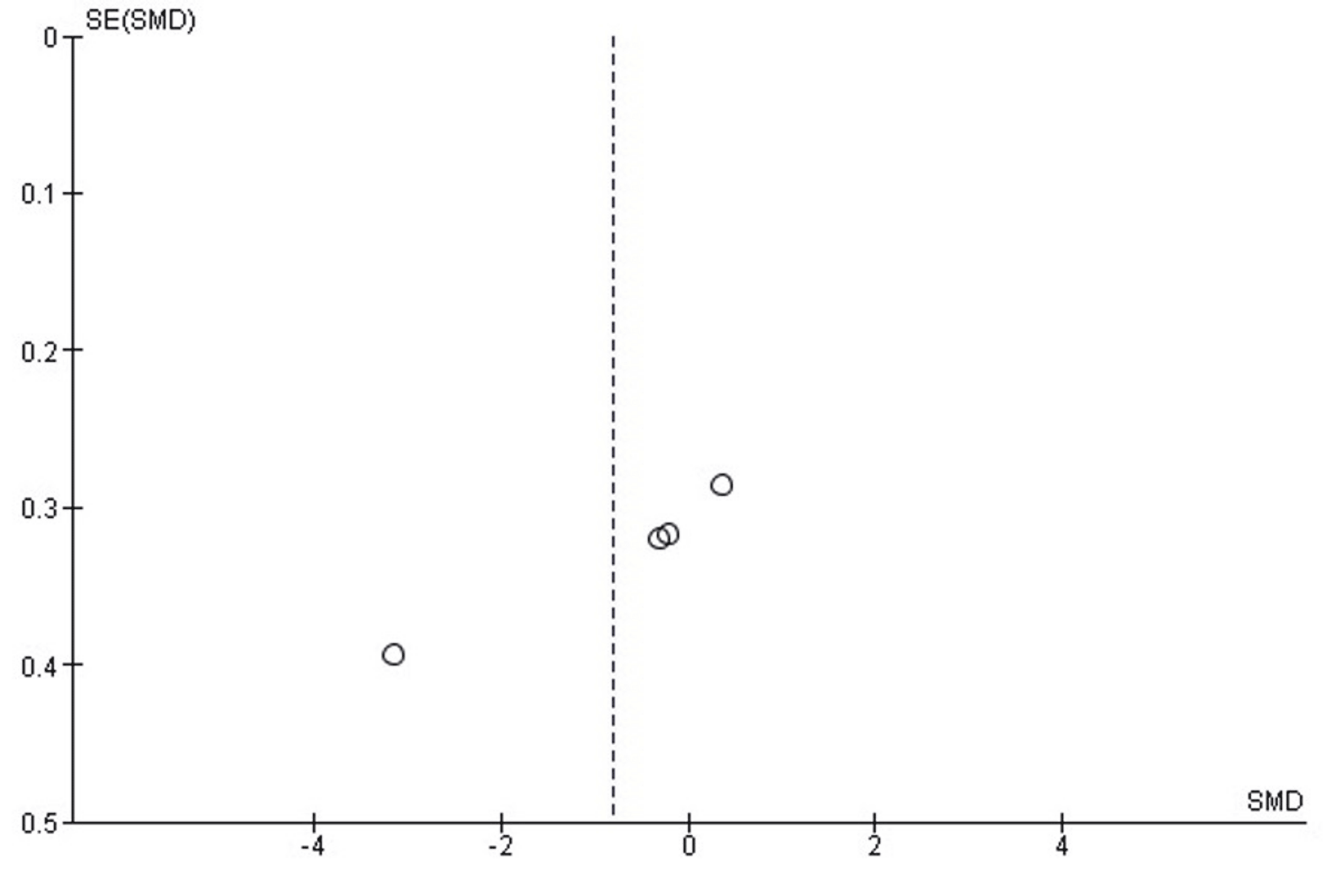
Funnel plot assessing publication bias for time-to-union comparison. SE: Standard Error; SMD: Standardized Mean Difference

The funnel plot (Figure 7) showed notable asymmetry, driven predominantly by a single small-sample outlier with a large negative effect. Despite this visual imbalance, the overall distribution did not suggest a coherent directional bias, and Egger’s regression test was not statistically significant (p > 0.05), indicating that meaningful publication bias is unlikely.

Comparison of Radiation Time Between SMP and TENS

**Figure 8:**
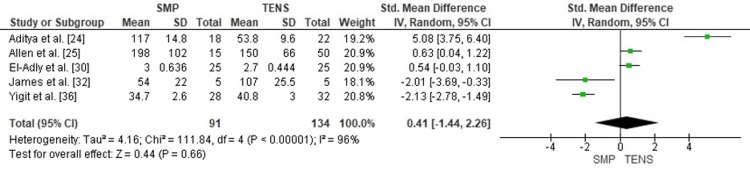
Forest plot comparing radiation time between submuscular plating and titanium elastic nailing. SMD: Standardized Mean Difference; CI: Confidence Interval Source: Refs [24,25,30,32,36]

A pooled comparison of intraoperative radiation exposure demonstrated no statistically significant difference between SMP and TENS (SMD = 0.41; 95% CI: -1.44 to 2.26; p = 0.66). Heterogeneity was extremely high (chi² = 111.84, df = 4, p < 0.00001; I² = 96%), indicating substantial variability in fluoroscopy duration caused by differences in case complexity, imaging protocols, and surgical experience across studies (Figure 8).

Publication Bias Assessment

**Figure 9:**
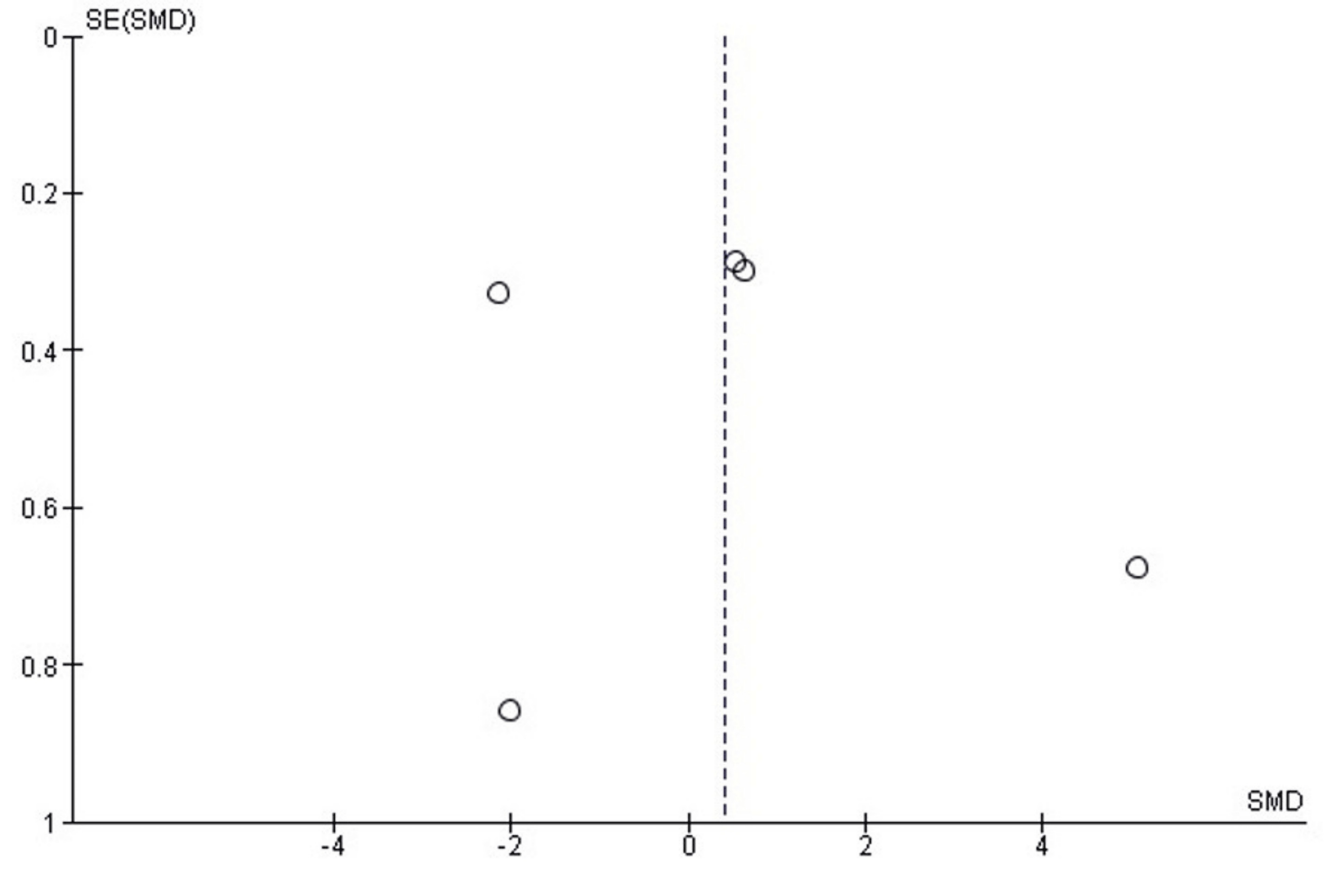
Funnel plot assessing publication bias for radiation time comparison. SE: Standard Error; SMD: Standardized Mean Difference

The funnel plot (Figure 9) showed considerable asymmetry, largely attributable to small-sample outliers with extreme effects. Despite this visual irregularity, there was no systematic directional pattern, and Egger’s regression test was not statistically significant (p > 0.05), suggesting that publication bias is unlikely to meaningfully affect the pooled estimate.

Comparison of Soft-Tissue Irritation Rates Between SMP and TENS

**Figure 10:**
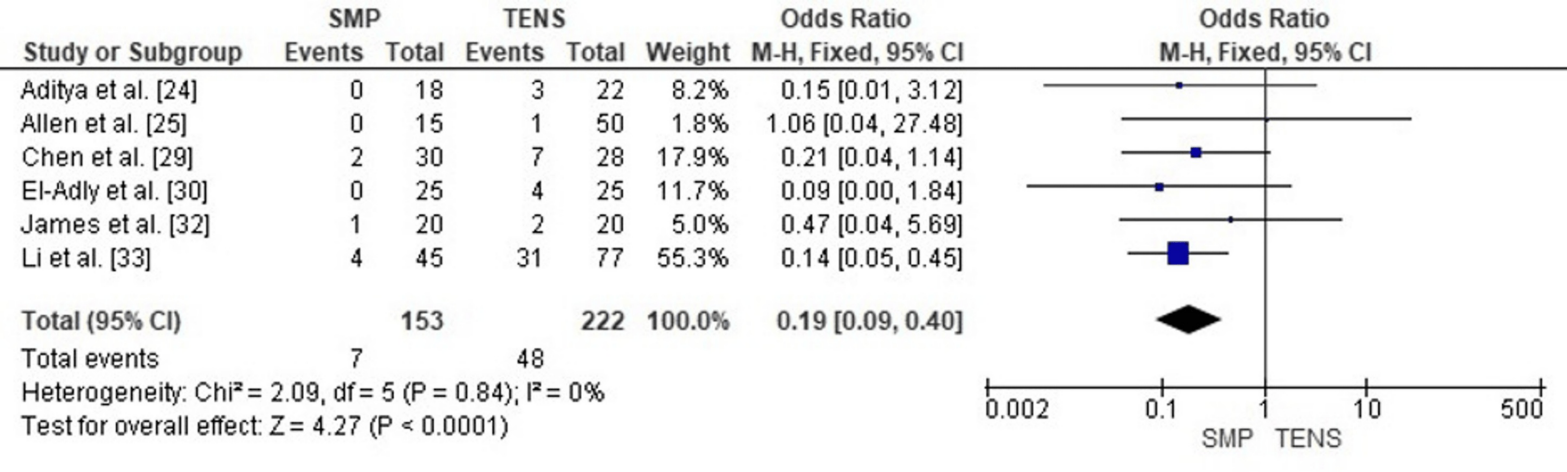
Forest plot comparing soft-tissue irritation rates between submuscular plating and titanium elastic nailing. OR: Odds Ratio; CI: Confidence Interval Source: Refs [24,25,29,30,32,33]

Soft-tissue irritation was significantly less frequent with SMP than withTENS (OR = 0.19; 95% CI: 0.09-0.40; p < 0.0001). Heterogeneity was negligible (chi² = 2.09, df = 5, p = 0.84; I² = 0%), indicating highly consistent findings across the included studies (Figure 10). The higher irritation rate with TENS is consistent with the prominence of nail ends at the metaphysis and the potential for local soft-tissue conflict.

Publication Bias Assessment

**Figure 11:**
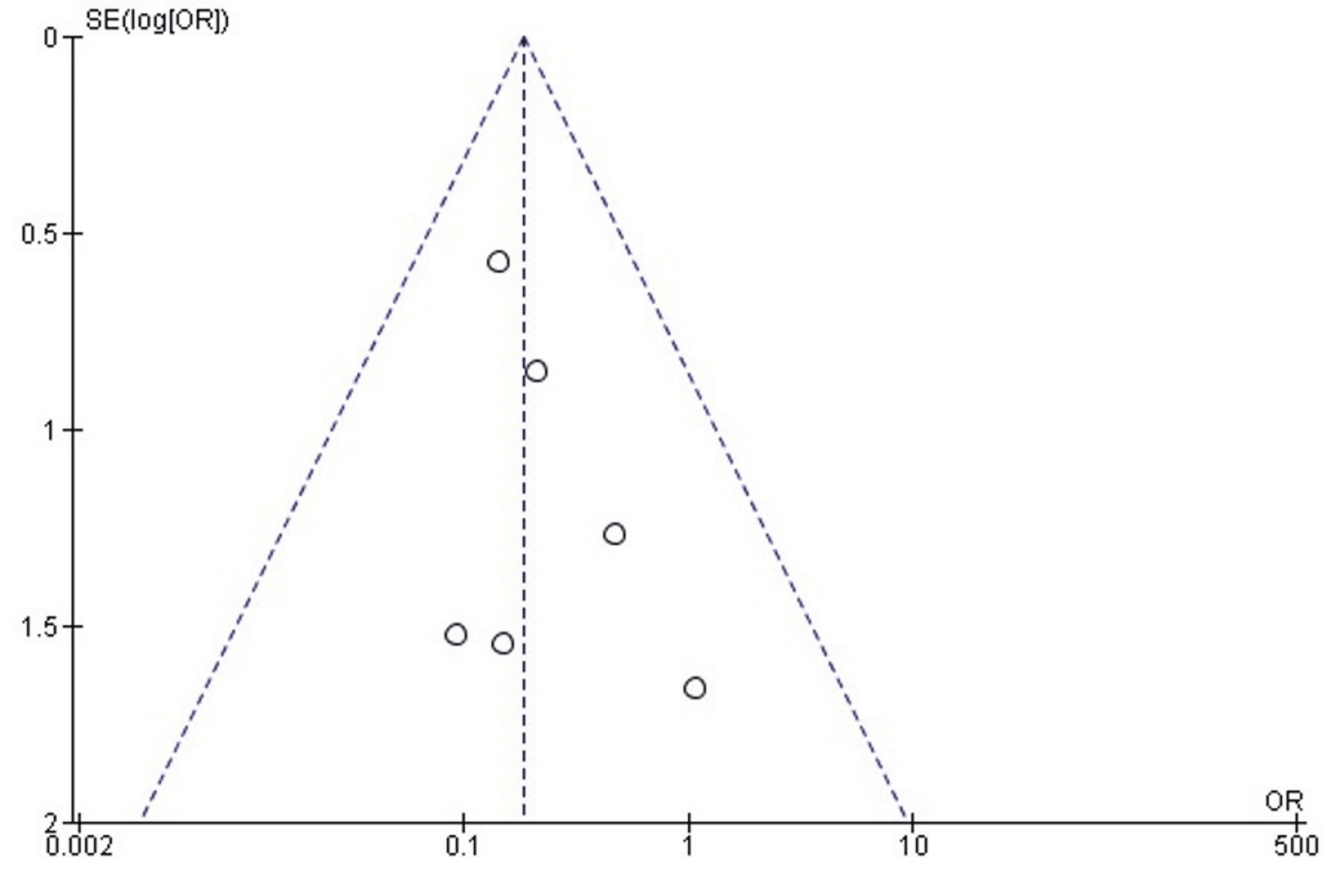
Funnel plot assessing publication bias for soft-tissue irritation comparison. SE: Standard Error; OR: Odds Ratio

The funnel plot (Figure 11) demonstrated a reasonably symmetrical distribution of study estimates, with no clustering pattern suggestive of small-study effects. Egger’s regression test was not statistically significant (p > 0.05), indicating that publication bias is unlikely to have meaningfully influenced the pooled results for soft-tissue irritation.

*Comparison of Unplanned Reoperation Rates Between SMP and *TENS

**Figure 12:**
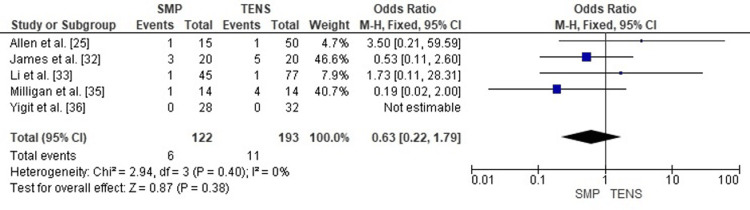
Forest plot comparing unplanned reoperation rates between submuscular plating and titanium elastic nailing. OR: Odds Ratio; CI: Confidence Interval Source: Refs [25,32,33,35,36]

The pooled analysis demonstrated no statistically significant difference in unplanned reoperation rates between SMP and TENS (OR = 0.63; 95% CI: 0.22-1.79; p = 0.38). Heterogeneity was negligible (chi² = 2.94, df = 3, p = 0.40; I² = 0%), indicating that the included studies were highly consistent in reporting comparable reoperation risks across both fixation methods (Figure 12). These results suggest that neither technique confers a clear advantage in terms of reducing the likelihood of unforeseen secondary surgery.

Publication Bias Assessment

**Figure 13:**
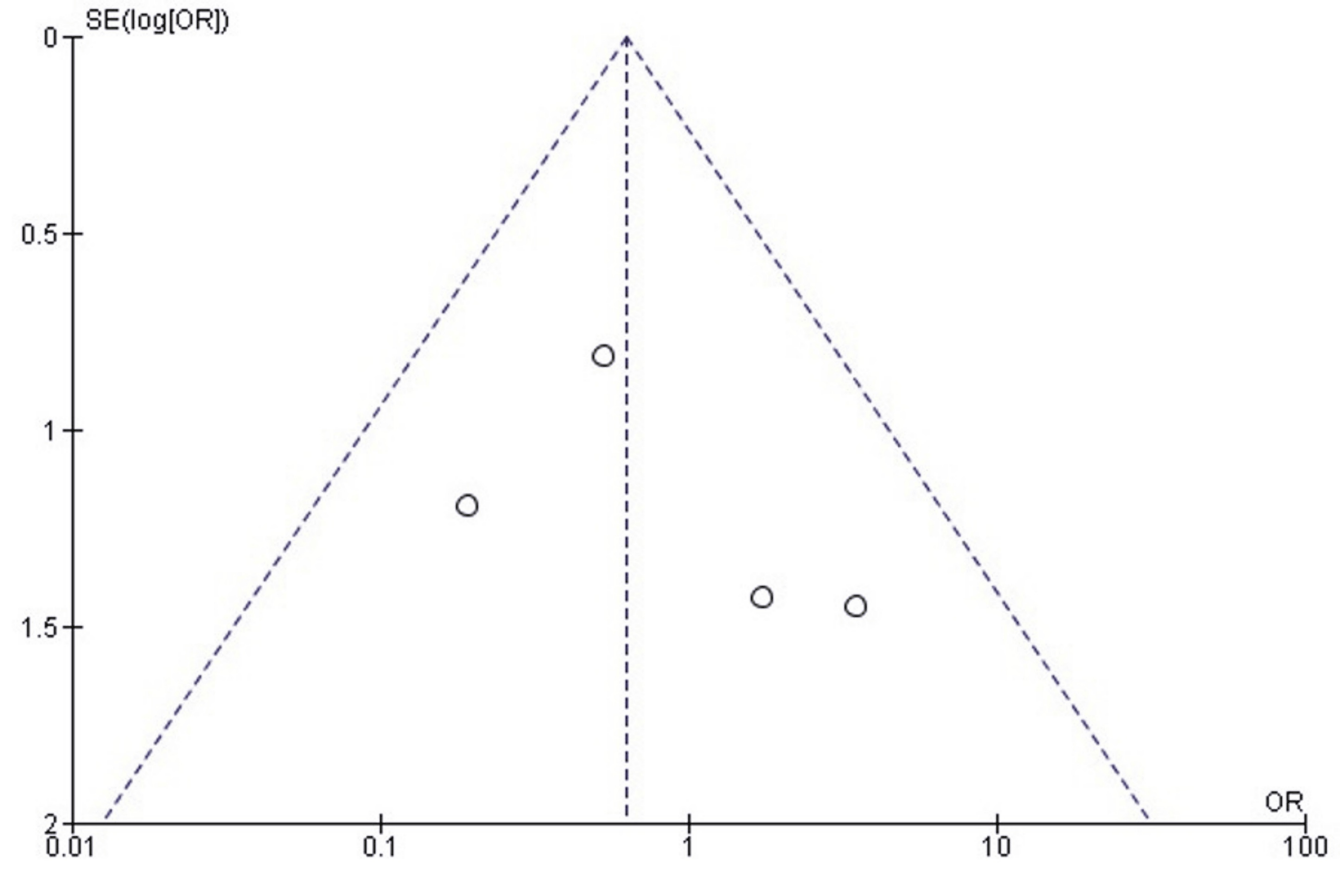
Funnel plot assessing publication bias for unplanned reoperation comparison. SE: Standard Error; OR: Odds Ratio

The funnel plot (Figure 13) showed a symmetrical distribution of effect estimates, with no evidence of small-study effects. Egger’s regression test was not statistically significant (p > 0.05), indicating that publication bias is unlikely to have influenced the findings for this outcome.

*Comparison of Excellent Flynn Outcomes Between SMP and *TENS

**Figure 14:**
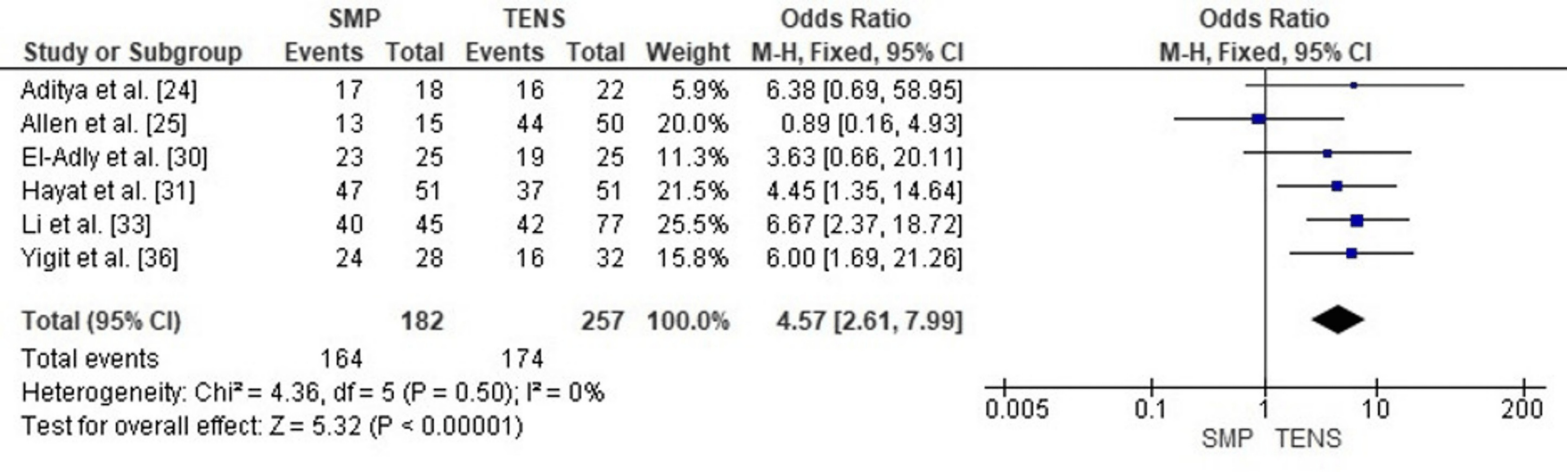
Forest plot comparing excellent Flynn outcomes between submuscular plating and titanium elastic nailing. OR: Odds Ratio; CI: Confidence Interval Source: Refs [24,25,30,31,33,36]

The pooled analysis demonstrated that SMP was significantly more likely to achieve excellent Flynn functional outcomes compared with TENS (OR = 4.57; 95% CI: 2.61-7.99; p < 0.00001). Heterogeneity was absent (chi² = 4.36, df = 5, p = 0.50; I² = 0%), reflecting strong consistency among all included studies (Figure 14). These findings indicate that SMP may offer a superior probability of achieving excellent postoperative functional results in pediatric femoral shaft fractures.

Publication Bias Assessment

**Figure 15:**
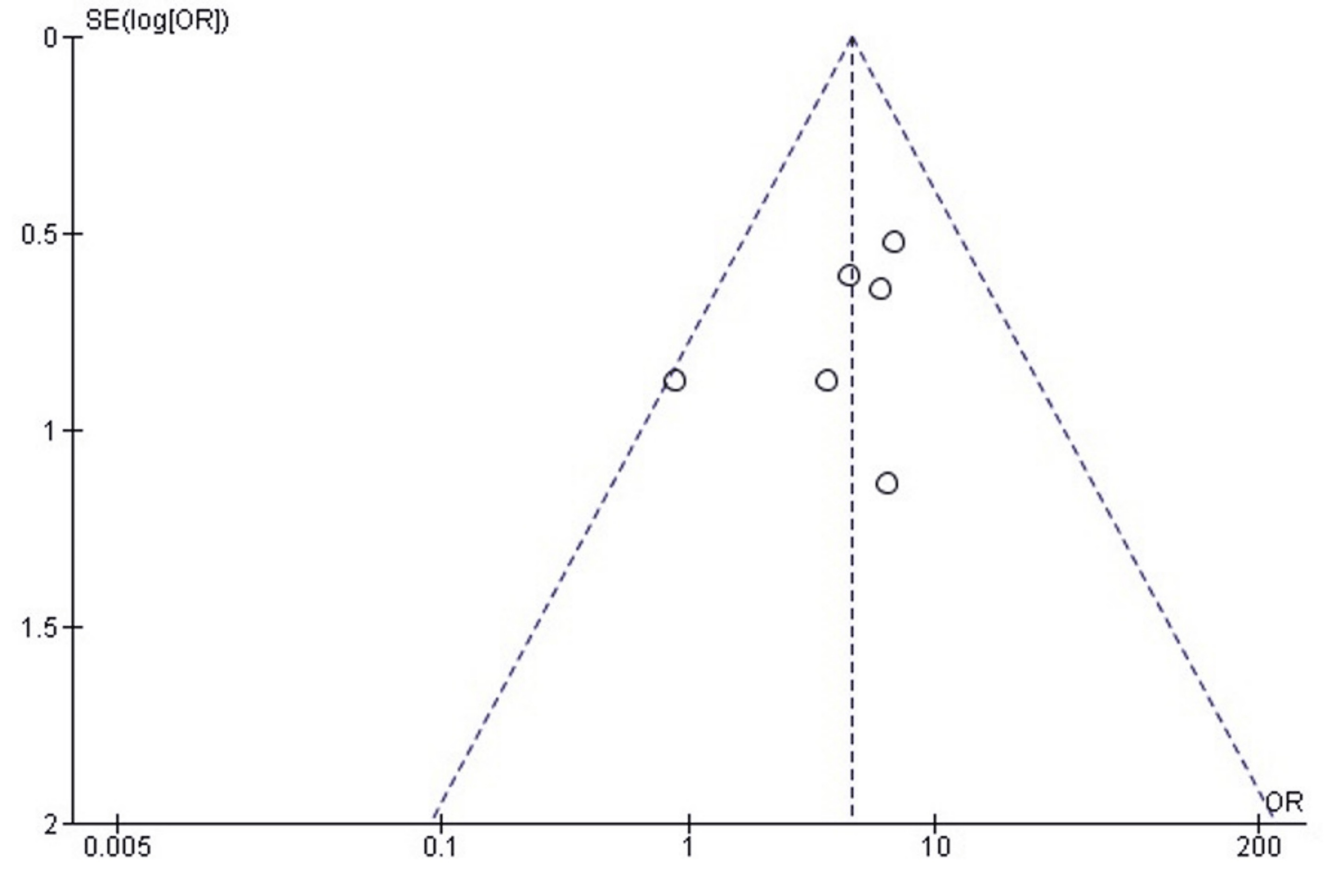
Funnel plot assessing publication bias for excellent Flynn outcomes. SE: Standard Error; OR: Odds Ratio

The funnel plot (Figure 15) demonstrated a generally symmetrical pattern without major outliers, and Egger’s regression test was not statistically significant (p > 0.05), indicating that publication bias is unlikely to have affected this outcome.

Comparison of Infection Rates Between SMP and TENS

**Figure 16:**
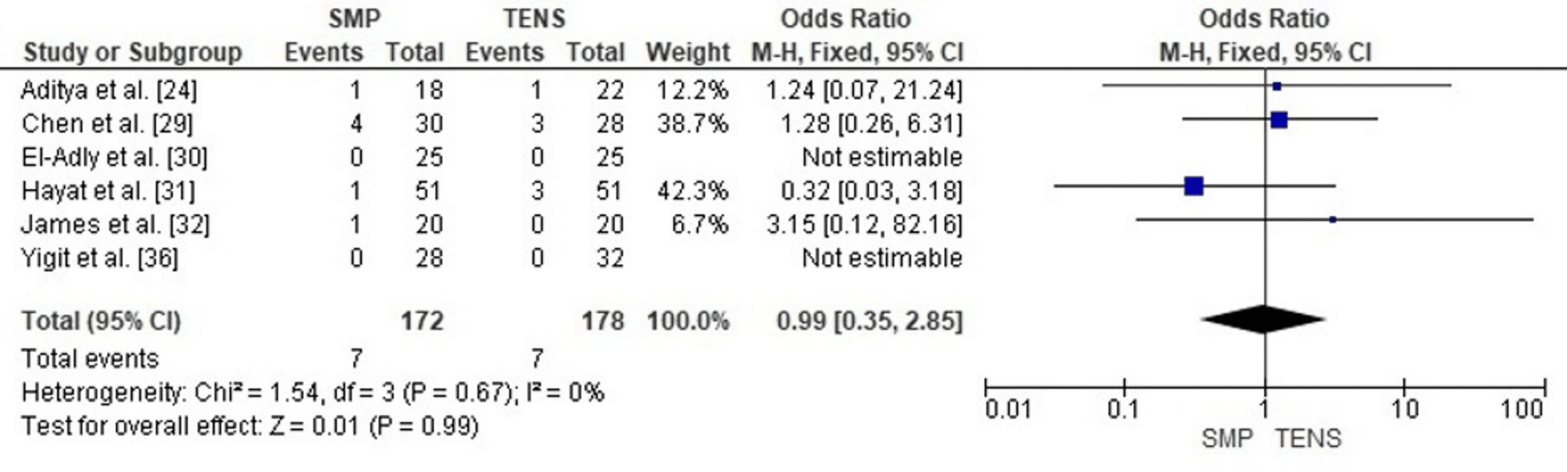
Forest plot comparing infection rates between submuscular plating and titanium elastic nailing. OR: Odds Ratio; CI: Confidence Interval Source: Refs [24,29,30,31,32,36]

The pooled analysis demonstrated no statistically significant difference in postoperative infection rates between SMP and TENS (OR = 0.99; 95% CI: 0.35-2.85; p = 0.99). Heterogeneity was absent (chi² = 1.54, df = 3, p = 0.67; I² = 0%), indicating a high level of consistency among the included studies (Figure 16). These findings suggest that both techniques carry a similarly low risk of infection in the management of pediatric femoral shaft fractures.

Publication Bias Assessment

**Figure 17:**
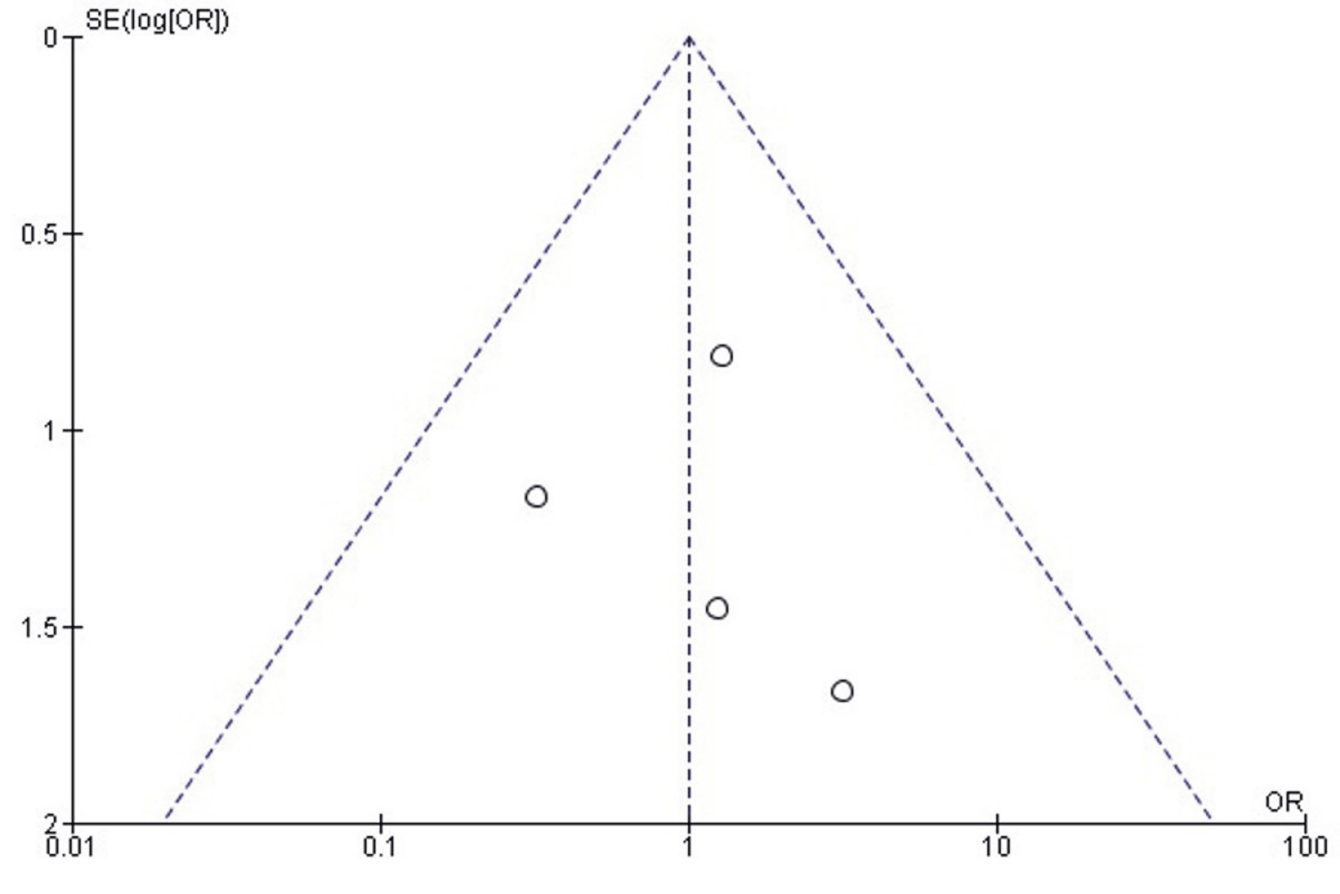
Funnel plot assessing publication bias for infection rate comparison. SE: Standard Error; OR: Odds Ratio

The funnel plot (Figure 17) showed a broadly symmetrical pattern with no substantial outliers, and Egger’s regression test was not statistically significant (p > 0.05). This indicates that publication bias is unlikely to have influenced the pooled estimate for infection rates.

Comparison of Time to Union Between TENS and EF

**Figure 18:**
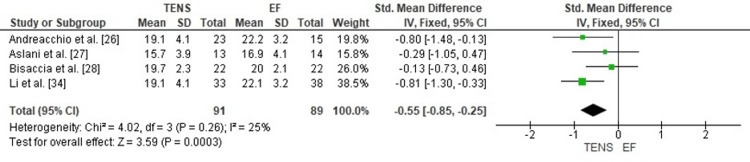
Forest plot comparing time to union between titanium elastic nailing and external fixation. SMD: Standardized Mean Difference; CI: Confidence Interval Source: Refs [26-28,34]

The pooled analysis demonstrated that TENS was associated with significantly shorter time to radiographic union compared with EF (SMD = -0.55; 95% CI: -0.85 to -0.25; p = 0.0003). A negative SMD indicates faster union in the TENS group. Heterogeneity was low (chi² = 4.02, df = 3, p = 0.26; I² = 25%), suggesting good consistency among the included studies (Figure 18). These findings indicate a clear advantage for TENS in achieving earlier fracture union.

Publication Bias Assessment

**Figure 19:**
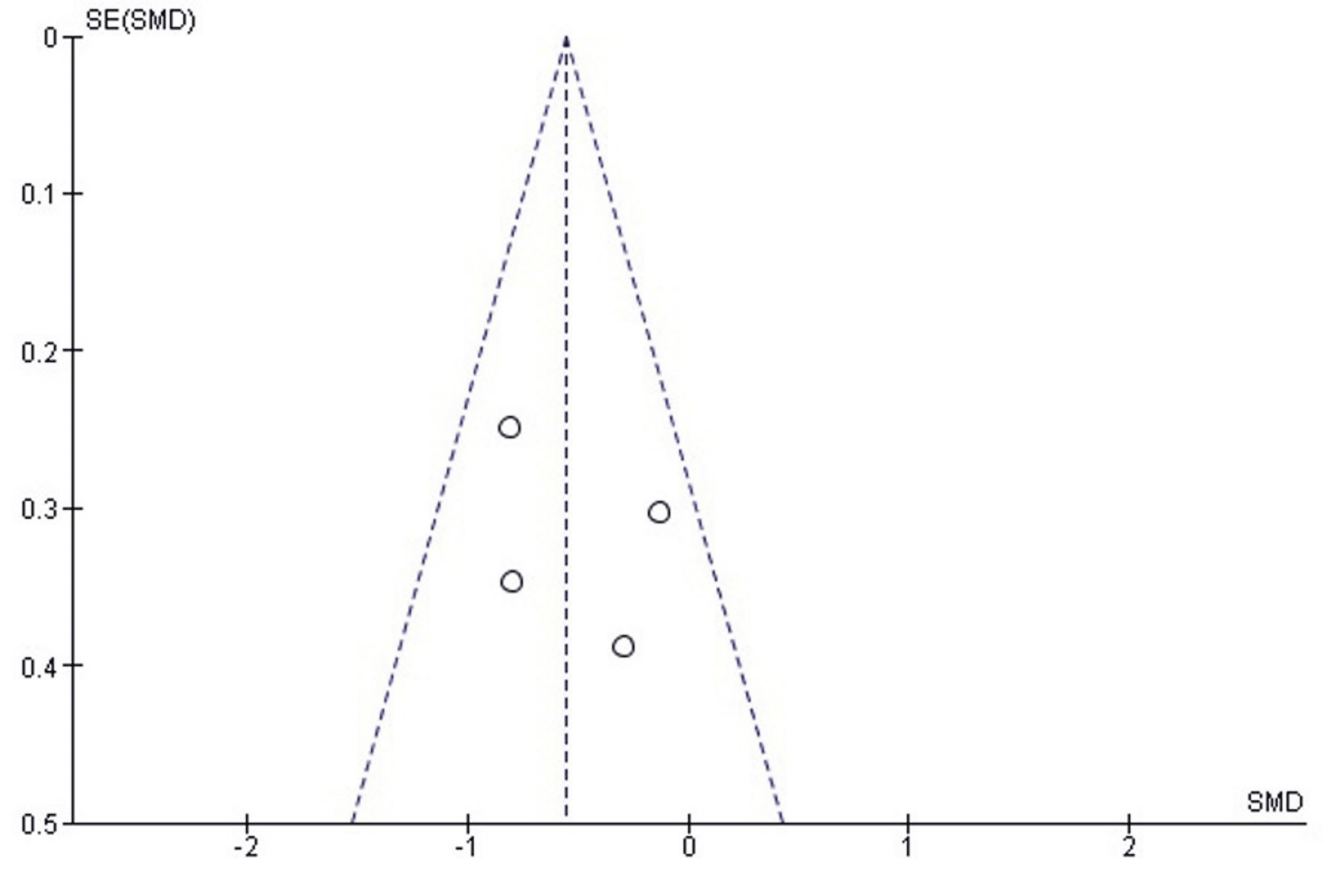
Funnel plot assessing publication bias for time-to-union comparison. SE: Standard Error; SMD: Standardized Mean Difference

The funnel plot (Figure 19) demonstrated a largely symmetrical distribution of effect sizes, with no major small-study effects. Egger’s regression test was not statistically significant (p > 0.05), indicating that publication bias is unlikely to have influenced the pooled estimate.

Comparison of Surgical Site Infection Rates Between TENS and EF

**Figure 20:**
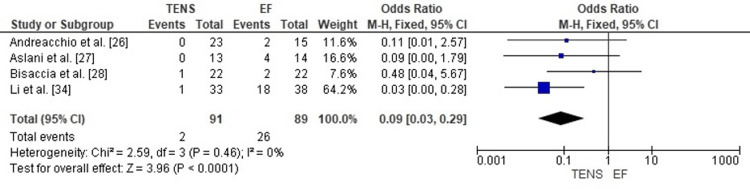
Forest plot comparing surgical site infection rates between titanium elastic nailing and external fixation. OR: Odds Ratio; CI: Confidence Interval Source: Refs [26-28,34]

The pooled analysis showed that TENS was associated with significantly lower surgical site infection rates compared with EF (OR = 0.09; 95% CI: 0.03-0.29; p < 0.0001). The odds ratio far below 1 indicates a strong protective effect of TENS against postoperative infection. Heterogeneity was absent (chi² = 2.59, df = 3, p = 0.46; I² = 0%), demonstrating excellent consistency among the included studies (Figure 20). These findings highlight a major advantage of TENS over EF in minimizing infectious complications in pediatric femoral shaft fracture management.

Publication Bias Assessment

**Figure 21:**
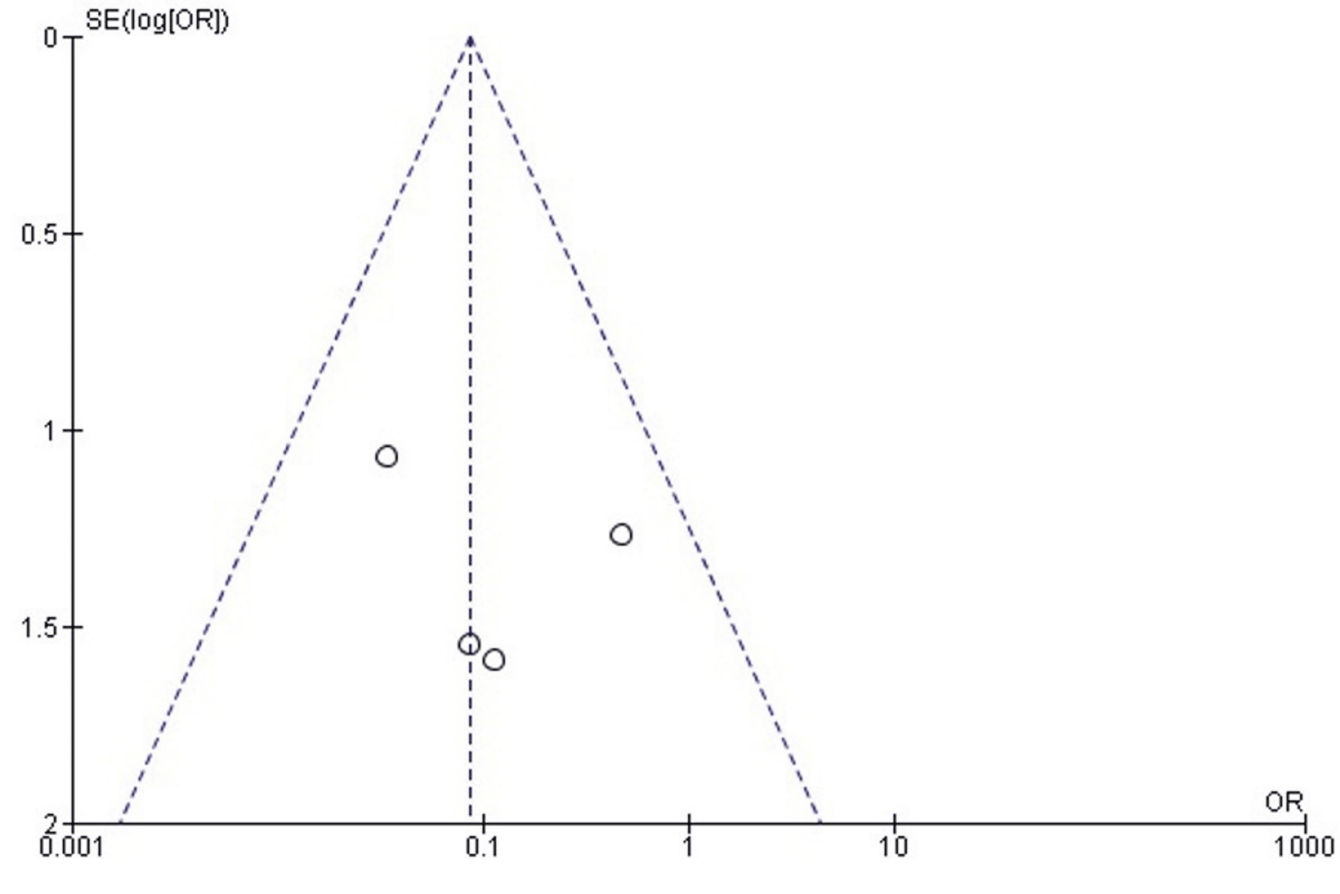
Funnel plot assessing publication bias for surgical site infection comparison. SE: Standard Error; OR: Odds Ratio

The funnel plot (Figure 21) displayed a symmetrical pattern without major outliers, and Egger’s regression test was not statistically significant (p > 0.05). This suggests that publication bias is unlikely to have influenced the observed protective effect of TENS on infection rates.

Comparison of Refracture Rates Between TENS and EF

**Figure 22:**
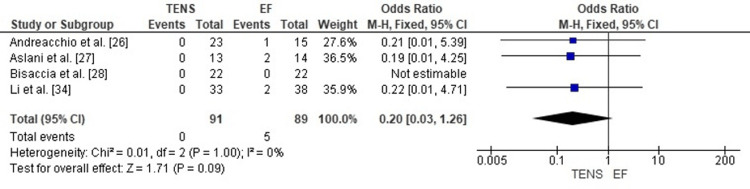
Forest plot comparing refracture rates between titanium elastic nailing and external fixation. OR: Odds Ratio; CI: Confidence Interval Source: Refs [26-28,34]

The pooled analysis demonstrated no statistically significant difference in refracture rates between TENS and EF (OR = 0.20; 95% CI: 0.03-1.26; p = 0.09). Although the point estimate favors TENS - with an 80% lower odds of refracture - the confidence interval includes 1.0, indicating that the difference is not statistically significant. Heterogeneity was absent (chi² = 0.01, df = 2, p = 1.00; I² = 0%), showing consistency across the included studies (Figure 22).

Publication Bias Assessment

**Figure 23:**
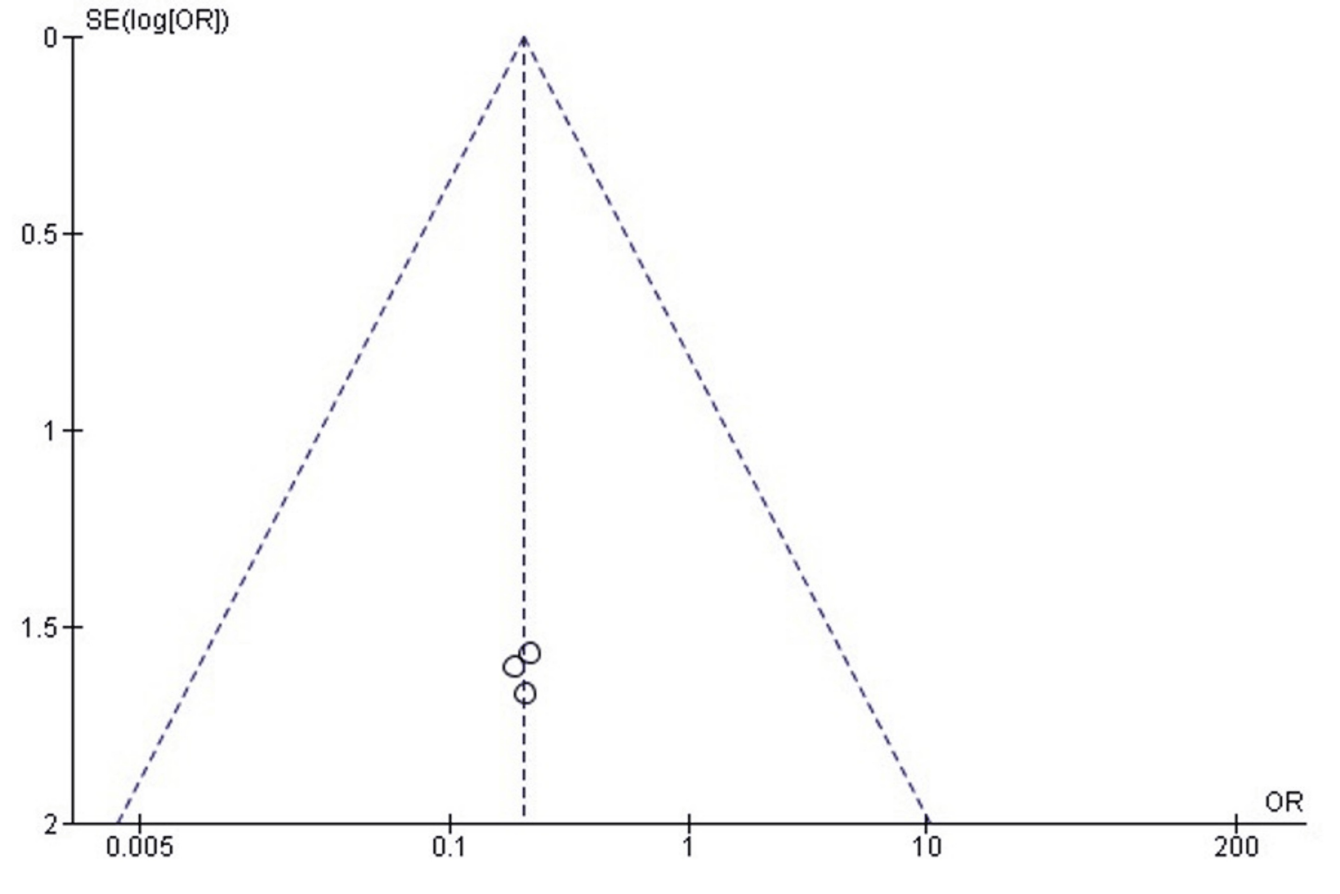
Funnel plot assessing publication bias for refracture comparison. SE: Standard Error; OR: Odds Ratio

The funnel plot (Figure 23) demonstrated a symmetrical distribution without noticeable small-study effects, and Egger’s regression test was not statistically significant (p > 0.05). This suggests that publication bias is unlikely to have influenced the refracture outcome.

Discussion

This systematic review and meta-analysis synthesised the comparative evidence for SMP, TENS, and EF in the management of paediatric femoral shaft fractures. Across all three modalities, union rates were high and long-term clinical outcomes were generally favourable, yet important differences emerged in perioperative efficiency, complication profiles, and functional recovery that are highly relevant to implant selection in daily practice.

When SMP was compared with TENS, a consistent trade-off between operative invasiveness and early function was observed. SMP required longer operative time and was associated with greater intraoperative blood loss in most cohorts, reflecting the need for wider exposure and submuscular tunnelling for plate insertion [24,25,29,30,32,33]. Aditya et al. [24] and Chen et al. [29] both reported substantially increased blood loss with SMP compared with elastic nailing, and similar trends were seen in other comparative series [25,30,33]. In contrast, TENS allowed shorter procedures with less soft-tissue trauma and reduced blood loss, highlighting its appeal as a minimally invasive option, particularly in standard length-stable fracture patterns [25,29,30,33].

Despite these perioperative differences, hospital stay and time to union were broadly equivalent between SMP and TENS. James et al. [32] reported similar median union times of approximately 12-13 weeks and comparable inpatient durations in both groups, and El-Adly et al. [30] and Hayat et al. [31] likewise found that most fractures in both SMP and TENS cohorts had united radiographically by 10-12 weeks. These findings suggest that fracture biology and reduction quality are stronger determinants of union than implant choice, provided that stable fixation is achieved [30-32].

The most consistent advantage of SMP over TENS is related to soft-tissue irritation and functional recovery. TENS was associated with higher rates of nail prominence and local irritation at entry points, often necessitating repeated outpatient visits or prompting earlier hardware removal [28,29,33]. Li et al. [33] reported implant irritation in more than 40% of TENS cases compared with less than 10% of SMP cases, while Bisaccia et al. [28] also highlighted nail-related discomfort in their TENS cohort. By contrast, the low-profile submuscular plate construct is concealed within the soft-tissue envelope and therefore avoids this particular source of morbidity, contributing to smoother early rehabilitation [28,29,33].

These differences translated into more favourable Flynn functional categories with SMP in several studies. Aditya et al. [24] reported excellent Flynn outcomes in more than 90% of SMP patients compared with approximately 73% of those treated with TENS. Similar patterns were demonstrated by Hayat et al. [31] and Li et al. [33], where SMP cohorts showed higher proportions of excellent scores, better early hip and knee motion, and earlier progression to full weight bearing. However, not all analyses showed a clear superiority of SMP. Allen et al. [25] and Milligan et al. [35] both documented more than 85% excellent or satisfactory Flynn results in both groups, with no statistically significant difference in overall functional grading. Taken together, these data indicate that both SMP and TENS can deliver high-quality functional outcomes when fracture selection is appropriate and reduction is anatomic, with SMP offering an incremental advantage in heavier children, length-unstable patterns, or when maximising early function is a priority [24,25,30-33,35].

In terms of safety, implant removal, infection, and unplanned reoperation rates were broadly similar between SMP and TENS. Decisions regarding hardware removal were mostly elective, guided by surgeon preference, parental expectations, or symptomatic implants rather than true implant failure [29,33,35]. James et al. [32] and Chen et al. [29] both reported low and comparable infection rates for SMP and TENS, with deep infection being rare in both groups. Milligan et al. [35] observed more unplanned returns to theatre in the TENS cohort, mainly due to nail migration or irritation, but this finding was not consistently reproduced in other series [25,29,32,33]. Radiation exposure also did not differ meaningfully between SMP and TENS, as fluoroscopy time appeared to be more dependent on surgeon technique and institutional workflow than on the choice of implant [24,25,30,32,36].

When TENS was compared with EF, the contrasts were more pronounced. TENS generally achieved faster or at least equivalent union with fewer complications. In children younger than eight years, Andreacchio et al. [26] reported shorter healing times with elastic nailing compared with EF. Aslani et al. [27] demonstrated similar union times in high-grade open fractures but faster functional recovery and earlier return to school in the TENS group. Li et al. [34] confirmed more predictable callus formation and more reliable progression of radiographic healing with elastic nailing, whereas EF showed a tendency toward delayed consolidation, likely influenced by pin-bone micromotion and variable frame stability [26,27,34].

Complication profiles strongly favoured TENS. EF was consistently associated with higher rates of pin-tract infection, soft-tissue irritation around pins, and refracture after frame removal [26-28,34]. Aslani et al. [27] reported pin-tract infection in more than one quarter of EF patients, and both Bisaccia et al. [28] and Li et al. [34] found that nearly half of EF cases experienced pin-site problems requiring ongoing wound care. Refracture after removal of the frame, while relatively infrequent, was observed by Andreacchio et al. [26] and Li et al. [34], particularly when frames were removed early or when residual stability was marginal. By contrast, TENS complications were dominated by nail irritation, which is usually minor and rarely compromises union or long-term function [26-28,34].

Functional outcomes also tended to favour TENS over EF. Aslani et al. [27] documented earlier recovery of knee range of motion and a quicker return to normal activities in the TENS group despite comparable union times. Bisaccia et al. [28] and Li et al. [34] similarly observed more reproducible limb alignment and fewer residual deformities with elastic nails, whereas EF occasionally resulted in mild angular deviations related to pin loosening, frame adjustments, or challenges maintaining reduction throughout the healing period [27,28,34]. Taken together, these data support the use of EF as a damage-control or niche solution for severe open injuries and polytrauma rather than as a routine definitive fixation method for standard diaphyseal fractures [26-28,34].

The present findings align with and extend the conclusions of existing systematic reviews. Prior meta-analyses have shown that SMP and TENS provide comparable union and limb-length outcomes, with SMP associated with fewer angular deformities and soft-tissue complaints but longer operative times and greater blood loss [11,12]. Similarly, a separate systematic review comparing flexible nailing and EF reported higher rates of infection and deformity with EF, suggesting that elastic nails should be preferred whenever feasible [21]. By integrating both SMP versus TENS and TENS versus EF comparisons in a single analysis, the current review provides a more complete hierarchy of options across the spectrum of fracture patterns and patient characteristics [11,12,21,24-36].

From a clinical standpoint, these results support a pragmatic, pattern-based approach to implant selection. TENS remains an excellent first-line option for most length-stable diaphyseal fractures in school-aged children because it offers efficient surgery, reliable union, and a favourable overall complication profile [24,25,29,30,32,33]. SMP is particularly attractive for heavier children, length-unstable or comminuted patterns, distal-third fractures, and high-energy injuries where enhanced mechanical stability and superior functional scores may outweigh the trade-off of a more invasive procedure [24,30-33,35,36]. EF should be reserved for severe open fractures, polytrauma, or circumstances in which rapid stabilisation is required and intramedullary or submuscular implants are not immediately feasible, with a clear understanding of the higher risks of pin-site infection, refracture, and cosmetic dissatisfaction [26-28,34]. Future guidelines and treatment algorithms could incorporate these findings to support more consistent, evidence-based decision-making in paediatric femoral fracture care [11,12,21,24-36].

Limitations

This review has several limitations that should be considered when interpreting the findings. Most included studies were retrospective comparative cohorts rather than high-quality randomised controlled trials, which introduces risks of selection bias, confounding, and unmeasured differences in patient or fracture characteristics [24-36]. There was substantial clinical and methodological heterogeneity across studies, including variation in fracture patterns, implant selection criteria, surgical techniques, postoperative protocols, and definitions of union and complications, which contributed to high statistical heterogeneity for some pooled outcomes. Functional scores and patient-reported outcomes were inconsistently reported and often limited to short-term follow-up, restricting the ability to compare long-term functional recovery and health-related quality of life. Finally, although efforts were made to assess publication bias, the number of studies for some outcomes remained modest, and small-study effects cannot be completely excluded.

## Conclusions

High union rates in pediatric femoral shaft fractures, SMP, TENS, and EF are all achieved, but with distinct trade-offs. Due to its shorter operative time, TENS is best suited for most length-stable diaphyseal fractures, lower blood loss, and a favorable complication profile, while in heavier children and length-unstable patterns, SMP offers greater mechanical stability and superior functional outcomes. Because of its higher infection and pin-site complication rates, EF should be reserved for selected severe open injuries and damage-control scenarios.
